# The Effect of Bioactive Aliment Compounds and Micronutrients on Non-Alcoholic Fatty Liver Disease

**DOI:** 10.3390/antiox12040903

**Published:** 2023-04-10

**Authors:** Camelia Munteanu, Betty Schwartz

**Affiliations:** 1 Department of Plant Culture, Faculty of Agriculture, University of Agricultural Sciences and Veterinary Medicine, 400372 Cluj-Napoca, Romania; 2 The Institute of Biochemistry, Food Science and Nutrition, The School of Nutritional Sciences, Robert H. Smith Faculty of Agriculture, Food and Environment, The Hebrew University of Jerusalem, Rehovot 76100, Israel

**Keywords:** liver, NAFLD, antioxidants, bile acids, AMPK

## Abstract

In the current review, we focused on identifying aliment compounds and micronutrients, as well as addressed promising bioactive nutrients that may interfere with NAFLD advance and ultimately affect this disease progress. In this regard, we targeted: 1. Potential bioactive nutrients that may interfere with NAFLD, specifically dark chocolate, cocoa butter, and peanut butter which may be involved in decreasing cholesterol concentrations. 2. The role of sweeteners used in coffee and other frequent beverages; in this sense, stevia has proven to be adequate for improving carbohydrate metabolism, liver steatosis, and liver fibrosis. 3. Additional compounds were shown to exert a beneficial action on NAFLD, namely glutathione, soy lecithin, silymarin, Aquamin, and cannabinoids which were shown to lower the serum concentration of triglycerides. 4. The effects of micronutrients, especially vitamins, on NAFLD. Even if most studies demonstrate the beneficial role of vitamins in this pathology, there are exceptions. 5. We provide information regarding the modulation of the activity of some enzymes related to NAFLD and their effect on this disease. We conclude that NAFLD can be prevented or improved by different factors through their involvement in the signaling, genetic, and biochemical pathways that underlie NAFLD. Therefore, exposing this vast knowledge to the public is particularly important.

## 1. Non-Alcoholic Fatty Liver Disease

The liver is a central organ that makes life possible for humans and which is at the center of vital metabolic functions. The health of the liver often reflects a person’s overall health. The liver tissue can be the target of different diseases, and all of them are able to change the functions of the liver. Nowadays, one of the most common causes of primary and chronic liver disorders is non-alcoholic fatty liver disease (NAFLD) [1], which is an important public health problem in different age groups [2]. NAFLD is defined as an excessive accumulation of fat, mainly in the form of triglycerides in the liver epithelial cells or hepatocytes [3]. The disease encompasses a wide range of liver disorders, from simple steatosis to non-alcoholic steatohepatitis (NASH), terminal liver failure, which can eventually lead to liver carcinoma [4], which can cause death [5]. If simple hepatic steatosis is not effectively treated, it may progress to cirrhosis, which may lead to liver failure and the development of liver carcinoma.

NAFLD is formed from fat deposits in the liver cells and is associated with metabolic syndrome, obesity, and oxidative stress. NAFLD constitutes a spectrum of liver diseases associated with collateral metabolic and cardiovascular disorders [6]. NAFLD is also characterized by atherogenic dyslipidemia, postprandial lipemia, and HDL lipoprotein dysfunction [7].

In the last decade, NAFLD has emerged as the most common cause of chronic liver disease in developed countries. NAFLD is a global epidemic that threatens human health. Its incidence rate reaches around thirty percent. Its prevalence increases significantly between 70 and 90% among people with obesity or type 2 diabetes [T2D] [7]. NAFLD is directly related to the metabolic syndrome: central obesity, hyperglycemia, type 2 diabetes, arterial hypertension, and hypertriglyceridemia, which are usual components of the metabolic syndrome and are also known risk factors for NAFLD [8].

The occurrence of NAFLD directly increases with the rising prevalence of obesity, metabolic syndrome (MetS), and type 2 diabetes (T2D). In recent times, the number of people with obesity has increased globally. The worldwide prevalence of obesity registered for NAFLD and NASH patients was 51 and 81%, respectively [9]. NAFLD prevalence fluctuates from 60 to 95% in obese people [10].

Diabetes is one of the fastest growing global health emergencies of the 21st century [11]. Around 463 million people worldwide have been diagnosed with diabetes in 2019, and a 51% increase is expected by 2045, raising the prevalence of diabetes to 700 million.

The association between T2D and NAFLD is well established and T2D is highly prevalent in NAFLD patients [12]. Dai et al. [13] analyzed the data of 24 studies which included 35,599 T2D patients and found a prevalence of 59.67% of NAFLD, which rose to 77.87% in those with T2D and obesity. The majority of the NAFLD patients with T2D fulfilled criteria for MetS, highlighting the relationship between these conditions in the metabolic risk continuum.

NAFLD is characterized by a wide variety of liver changes, ranging from simple steatosis to non-alcoholic steatohepatitis (NASH), cirrhosis, and liver carcinoma. NASH is described as steatosis combined with inflammation and thus has become the second leading liver disease that leads to liver transplantation in the US [14]. Approximately one-third of US adults who have NAFLD also have NASH, and 30% of these individuals have the potential to progress to advanced cirrhosis, hepatocellular carcinoma, and liver-related mortality [15]. The pathogenesis of NAFLD/NASH is complicated and involves lipid accumulation, insulin resistance, inflammation, and fibrogenesis. During the progression of NAFLD, oxygen-containing free radicals (ROS) are activated and cause oxidative stress.

**Figure 1:**
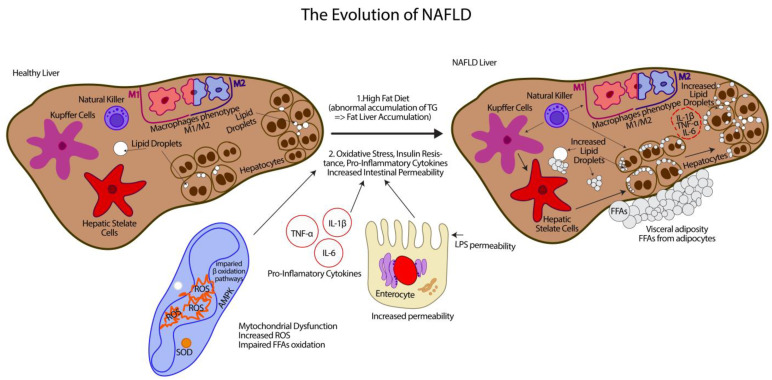
Liver damage in NAFLD.

The biological mechanism of the onset of basic steatosis and progression to liver disease is not fully understood, which is likely due to a number of factors that manifest in addition to genetic predisposition. The central concept of NAFLD is the “parallel hits” hypothesis [16], which was developed from the two-hit theory proposed by Day et al. in 1998 [17]. The two-hit theory asserts that a high-fat diet or diabetes-induced steatosis (the first hit) will make the liver more susceptible to other risk factors associated with oxidative stress and cause severe lipid oxidation (the second hit) (Figure 1).

In addition, insulin resistance has been shown to facilitate the progression of NAFLD to NASH [18]. Hepatic lipid overload and/or hyperinsulinemia-driven de novo lipogenesis increases lipid peroxidation, which results in the production of reactive oxygen species (ROS).

From this, the “two hits” hypothesis can be put forward [17]: the first hit is determined by an abnormal accumulation of triglycerides (TGs) and causes fat accumulation in the liver (steatosis), and the second hit is mainly caused by oxidative stress and triggers the progression of steatosis to NASH (Figure 1). As mentioned, it includes oxidative stress, insulin resistance, the secretion of pro-inflammatory cytokines, increased intestinal permeability, and obesity, which are identified as the main factors involved in the pathogenesis of NAFLD [16]. These factors, accompanied by oxidative stress, can promote intrahepatic fat accumulation and lipotoxicity, and develop inflammation, liver cell apoptosis, and fibrogenesis responsible for disease progression (Figure 1) [19].

A current view has recently been developed and is based on a more complex “multiple parallel hits hypothesis”, which includes a wide spectrum of parallel hits, including insulin resistance, oxidative stress, genetic and epigenetic mechanisms, environmental elements, cytokines, and changes in the microbiota. The theory of multiple parallel hits states that the effect in the development of NAFLD is more comprehensive and includes more diverse factors than a simple effect of one or two factors [20].

With regard to treatment, lifestyle intervention is the only approach to NAFLD. Thus, there is still no accepted effective drug treatment. The basis for the effective treatment of NAFLD is the identification of various components that intervene in each of the processes to prevent the progression of the disease. 

Human survival is associated with the consumption of various nutrients required for life, which consist of macronutrients such as carbohydrates, lipids, amino acids, dietary fiber, and micronutrients such as minerals and vitamins. In addition to these common nutrients, there are additional components in the diet such as coffee (rich in caffeine) and tea (comprising tea polyphenols), forming an important part of our daily diet. 

Dietary components reach the digestive system for the sake of digestion and absorption and then they are transformed into small molecule metabolites. Intestinal microorganisms further decompose nutrients into smaller units, such as bile acids (BAs) [21], short-chain fatty acids (SCFAs) [22], free fatty acids (FFAs) [21], and generate a wide variety of biological responses. All these processes may affect the intestinal microbiota and therefore may impinge on intestinal absorption, dietary energy, BAs metabolism, and finally may affect the intestinal permeability. Gut microbiota strains widely fluctuate from one individual to the other. The identity of the microbiome population and their quantity are highly dependent on the host genotype, on the host nutritional practices, and others [23]. 

Immune cells such as Th1/Th2 lymphocytes and T regulatory cells (T regs), macrophages, and natural killer cells play key roles in the pathogenesis of NAFLD. Specifically, hepatic macrophages, which consist of resident Kupffer cells and recruited bone marrow-derived macrophages, are the major cells that produce inflammatory mediators, such as tumor necrosis factor (TNF)-α and interleukin (IL)-1β, causing systemic insulin resistance followed by NAFLD and, ultimately, NASH [24]. In tissues, macrophages undergo maturation via specific pathways following their stimulation by different triggers, leading them to obtain specialized functional phenotypes. Toll-like receptors (TLRs), such as lipopolysaccharide (LPS) and interferon gamma (IFN-γ), are ligands that stimulate the conventional activation of M1, while IL-4/IL-13 stimulate the activation of M2 [25]. The dysregulation and polarization of M1/M2 macrophages can lead to chronic inflammation, infection, cancer, obesity, and their associated disorders such as NAFLD [26]. A novel mechanism that may regulate the M1/M2 balance relies on apoptotic effects of M2 Kupffer cells toward their M1 counterparts was previously published [27]. Wan et al. suggested that, by promoting M2-induced apoptosis of M1 Kupffer cells, one may prove a relevant strategy to limit high fat-induced inflammation and hepatocyte injury in NAFLD (Figure 1). Many food-derived molecules are associated with M1 and/or M2 activation. Depending on the progressive stages of NAFLD, NK cells that belong to innate immunity are distinct in phenotypes and frequency. They are also involved in inflammatory processes, hepatic steatosis, and fibrosis [28]. In the current review, we will focus on identifying aliment compounds, micronutrients, or promising bioactive nutrients that may interfere with NAFLD advance and will finally affect the disease progress. We used relevant information from many research articles and reviews by accessing some international databases over a fairly long period of time, namely 1963–2022. However, most studies focused on the next coverage period of 2005–2022. In this way, we will reach our final goal. In this search, we used specific phrases in order to obtain information that was as objective as possible. Additionally, some words such as liver, NAFLD, antioxidants, bile acids, AMPK, sweeteners, cannabinoids, and finally caffeine helped us significantly in gathering information and stabilizing the final organization of the review. This review was divided into the general presentation of NAFLD pathology; clinical information regarding nutrients with bioactive potential; and the presentation of different enzymes, which have influenced the progression and development of this pathology. All these correspond to 20 issues.

The liver has its own immunity. The components that belong to innate immunity and influence the pathology of NAFLD are represented by Kupffer cells, hepatic stellate cells, and natural killers. Additionally, besides these, there are macrophages which, depending on the situation, can change their M1/M2 phenotype. According to the “two hits” hypothesis that sustains the NAFLD, the abnormal accumulation of triglycerides (TGs) causes fat storage in the liver (steatosis), and oxidative stress can trigger the progression of steatosis into NASH. In order to argue in favor of this hypothesis, there is a variety of cellular, molecular, and signaling changes. They include oxidative stress, insulin resistance, the secretion of pro-inflammatory cytokines, and increased intestinal permeability.

## 2. Potential Bioactive Nutrients That May Interfere with NAFLD

### 2.1. Dark Chocolate

One of the richest foods in bioflavonoids (flavonols, polyphenols, and theobromine) is dark chocolate (DC) [29]. DC has the highest level of antioxidants compared to other food sources [30]. Besides nutrients such as saturated fat (60%), monounsaturated fat (35%), and linoleic acid (3%), chocolate contains important minerals such as potassium and magnesium [31] as well as cocoa, which is the main ingredient in chocolate [32]. Cocoa and some of its derivatives are a very complex food [33] and a rich source of antioxidants flavonoids, catechin, and epicatechin [34]. The potential health benefits of chocolate consumption have mainly been discovered recently [35]. Several recent studies have suggested that DC may have positive and changing effects on the lipid profile, reducing total and LDL cholesterol levels and increasing HDL levels [36].

Several studies have indicated that cocoa from chocolate may contribute to protective effects through the effect of the many beneficial components they contain such as minerals, antioxidants [37], and especially polyphenolic compounds (antioxidant flavonoids), which exhibit anti-inflammatory and antithrombotic activities. The beneficial metabolites may all contribute to their protective effect [38]. Furthermore, these can improve insulin resistance through reducing oxidative stress, improving endothelial function, and/or altering glucose metabolism [39]. A previous study on a rat model induced with alcoholic steatohepatitis showed that cocoa supplementation had a beneficial effect on the disease by reducing the accumulation of fat in the liver, reducing inflammation, and reducing cell necrosis [40]. Recent studies have mainly argued the protective context of cocoa or chocolate consumption and different health indicators [41]. The results of these studies affirmed that cocoa and DC intake can reduce oxidative stress, stimulate brain function, fight cancer cells, improve blood circulation, improve the mood, memory, immune system, and even protect the heart [42] and the liver tissue [43].

Therefore, our assumption to administrate dark chocolate may also have an adequate potential in the prevention and control of NAFLD. Support for this assumption is provided in a recent study among NAFLD patients in which they wanted to evaluate the effect of constant consumption of 30 g per day of dark chocolate (DC) containing 83% cocoa for 12 weeks on blood lipid profile, on fasting blood sugar levels, on enzyme levels in the liver, as well as the inflammatory status and antioxidants among patients suffering from NAFLD [44]. The findings of the above study demonstrate that the consumption of DC in the amounts indicated above causes a decrease in weight, BMI, and aspartate aminotransferase (AST) levels in the serum of NAFLD patients. The therapeutic effects of DC as a protective substance for the liver are still not well understood. According to the results obtained in the study, a daily dose of 30 DC (83%) for 12 weeks in patients with NAFLD resulted in an important reduction in BMI and body weight. The mechanism of NAFLD development is related to the existence of overweight and obesity, as well as the place of fat storage in the body (abdominal). Many studies that have investigated the effect of DC on body weight revealed conflicting results. Similar to the results of the aforementioned study [44], Massolt and his coworkers [45] found that the effects of DC consumption (85% cocoa) comprised appetite suppression and possibly lost-weight gain after eating 30 g of chocolate for the 12 subjects. It appears that DC can suppress lipid synthesis and grow lipolysis in adipose tissue, likely by enhancing the bioavailability of nitric oxide (NO) and increasing glucose uptake, intensification of fatty acid catabolism, and glucose oxidation. Moreover, in another study [46], it was found that cocoa is effective in reducing abdominal fat tissue in rats, possibly by changing genes for enzyme expression and transport molecules involved in fatty acid synthesis and thermogenesis in the liver and white adipose tissue (Table 1).

The results of the study of Alavinejad et al. [44] confirmed that the administration of DC for 12 weeks at a dose of 30 g per day can increase the levels of high-density lipoprotein cholesterol (HDL-C) in the serum of NAFLD patients. However, the serum levels of cholesterol such as toral cholesterol (TC), low-density lipoprotein (LDL), and very low-density lipoprotein (VLDL) were not affected after DC supplementation (Table 1). It is clear that postprandial hepatic lipid metabolism may be altered in patients with NAFLD. Furthermore, the suppression of LDL oxidation may be key to its anti-atherogenic attribute. The findings of Alavinejad et al. [44] are consistent with those reported by Hamed et al. [47], who showed that seven days of DC consumption increased serum HDL levels by 9% in 28 healthy volunteers. Another study [48] observed that the daily consumption of 45 g of chocolate rich in flavonoids for 4 months registered a notable rise in serum HDL levels in type 2 diabetes (T2DM) pathology. Additionally, in another study [49], they observed the effects of cocoa supplementation (36.9 g dark chocolate bar and 30.95 g cocoa powder drink) on the lipid profile in healthy subjects for 6 weeks. It can be noted that cocoa components, such as flavonols, include epicatechin, catechin, and procyanidins. These flavonols exhibit anti-inflammatory properties, which can regulate TNF-κB gene expression and reduce inflammatory biomarkers [50] and ROS production.

Elevated levels of liver enzymes (trans-aminases) such as AST and ALT in serum are known markers of NAFLD and liver diseases [51]. In the study by Alavinejad et al. [44], DC consumption for 12 weeks significantly reduced serum AST levels in NAFLD patients. Support for the above study was published by the group of McKim et al. [52], who found that the administration of cocoa extract (400 mg/kg per day) continuously for one month stimulates liver functions together with a notable decrease in fat accumulation, inflammation, and necrosis in early liver injuries caused by administering alcohol in a laboratory model in mice [52].

### 2.2. Cocoa Butter

Chocolates and cocoa-based products are praised for their “health benefits”, due to their polyphenol content. However, the fat content in chocolate has a supposed connection to the health benefits of chocolate [53]. Palmitic, stearic, lauric, and myristic acids are considered saturated fatty acids, oleic acid is a monounsaturated fatty acid, while linoleic acid is a polyunsaturated fatty acid. In cocoa butter (CB), there are fats that are naturally found in the cocoa beans. CB, also called theobroma oil, is a light-yellow vegetable fat that comes from the cocoa bean and is responsible for the melting properties of chocolate. As mentioned, cocoa butter (CB) consists mainly of palmitic fatty acids (C16:0), stearic acid (C18:0), oleic acid (C18:1), and linoleic acid (C18:2), with low levels of lauric acid (C12: 0) and myristic acid (C14:0) for cocoa butter and a fatty acid profile of approximately 60% saturated fat, 35% monounsaturated fat, and 1% polyunsaturated fat [54]. Although saturated fat is generally thought to adversely raise total cholesterol and LDL cholesterol levels, early studies also suggested that stearic acid may be hypo-cholesterolemic [55]. CB stearic acid can turn in the liver into oleic acid, a monounsaturated fatty acid, wherein oleic acid lowers cholesterol levels (LDL) and increases cholesterol levels (HDL) (Table 1). In addition, CB is a suitable source of Vitamin E [56].

A recent study [57] on rats fed an ethanol-enriched diet that causes alcoholic liver damage in rats evaluated the role of saturated fats from cocoa butter (plant source) compared to lard (animal source). After 8 weeks of feeding, plasma aspartate aminotransferase (AST) and alanine aminotransferase (ALT) activity, liver triglyceride (TG) levels, intercellular adhesion molecule (ICAM)-1 levels, liver cytochrome P450 2E1 (CYP2E1), and liver protein expression were recorded. Interleukin (IL)-1β was significantly increased in ethanol-fed rats. In addition, liver histopathological scores of fatty changes, inflammatory cell infiltration, and atrophy and necrosis were significantly altered. However, fatty changes were significantly inhibited only in rats fed ethanol and cocoa butter as along with inflammatory cell infiltration, degeneration, and necrosis in the liver. In addition, plasma ICAM-1 and hepatic tumor necrosis factor (TNF)-α, IL-1β, IL-6, and IL-10 levels were significantly lower in rats fed ethanol and cocoa butter. Moreover, correlation analysis showed that liver histopathological scores of atrophy and necrosis were significantly positively correlated with erythrocytic oleic acid (C18:1) and negatively correlated with linoleic acid (C18:2). In conclusion, cocoa butter protected the liver from lipid accumulation and inflammation in rats fed chronic ethanol (Table 1).

In summary, the daily recommendation of cocoa butter consumption is one tablespoon of cocoa butter. This tablespoon contains 8 g of saturated fatty acids, including 4 g of stearic acid, 4 g of monounsaturated fatty acids, and 0.5 g of polyunsaturated fatty acids (mostly omega-6) [58].

### 2.3. Peanut Butter

Peanuts originated in Central America and were afterward spread out to other regions of the world [59]. Today, peanuts are among the most significant legume crops in the world and they are also considered as oilseeds due to their high lipids matter. In addition to oil, a great variety of peanut products such as flour, peanut butter, milk, and more have been developed [60].

Peanuts contain about 50% monounsaturated fatty acids (MUFAs), 33% polyunsaturated fatty acids (PUFAs), and 14% saturated fatty acids. The greatest quantity of fatty acids found in peanuts is represented bytriglycerides, which are from 93.3% to 95.5% of the total fatty acid weight [60]. The standard peanut varieties have an oil profile containing about 52% oleic acid and about 27% linoleic acid. Roasted peanuts contain about 21.5% carbohydrates with starch being the main carbohydrate [61]. Peanuts are considered to have a low glycemic index [62], and the consumption of peanuts or peanut oil is linked with a low risk of cardiovascular disease and may ameliorate the lipid profile [63]. A high intake of peanuts or peanut butter has been linked with protection against the development of diabetes [64]. Peanut butter and even peanut oil, in combination with a slimming diet, allow for maintaining a stable body weight in the long term [65]. Despite these adequate effects of peanut consumption, their effect on fatty liver disease has hardly been studied.

It is clear that some of the health properties of peanuts are related to their nutritional composition, particularly their fat profile. The fat content in conventional varieties is about 50% MUFAs and about 25% PUFAs. Dietary recommendations place great importance on consuming up to 20% of the total daily caloric intake from MUFA oils such as those found in olive oil. One of the main fats in peanut butter is oleic acid. Oleic acid contributes to balancing cholesterol levels, blood sugar, and blood pressure. Managing these levels in the body can lower the risk of heart disease [66].

Oleic acid has also been shown to reduce the body’s resistance to insulin, a condition that raises blood sugar and leads to diabetes. Research shows that the omega-6 content of peanut butter may have the same effect as well. Peanut butter also contains omega-6. This fatty acid decreases low cholesterol (LDL) and increases protective cholesterol (HDL) (Table 1). Additionally, peanuts are a natural source of arginine, an amino acid that may prevent cardiovascular disease by promoting adequate blood vessel function [67].

Peanuts are an excellent source of antioxidants such as manganese, Vitamin E, and B vitamins. These compounds work to prevent and repair cell damage, and this effect can reduce the risk of chronic diseases such as NAFLD [59,61]. Due to the high content of antioxidants and vitamins, peanut butter can improve antioxidant activity in the liver and promote its health. Along with peanuts, peanut butter is also a balanced source of protein that is especially useful for NAFLD patients, as they have many dietary restrictions. One of the most powerful antioxidants in peanut butter is coumaric acid [68]—and a study found that its activity is increased by 22% if the peanuts are roasted before turning them into butter. Peanuts also contain resveratrol, an antioxidant that has been shown to have anti-cancer effects and may lower the risk of obesity, heart disease and cognitive decline. In addition, foods rich in healthy fats, proteins, and fiber—such as peanut butter—take longer to digest, which can contribute to a longer feeling of satiety and reduce the risk of overeating [59].

The recommended daily dose of peanut butter is 2 tablespoons of natural peanut butter (about 28 g). This dose is equivalent to 160–200 calories, it is therefore true that this is a spread high in fat and calories, but at the same time, eating peanut butter will provide a long-lasting feeling of satiety, thus preventing the phenomenon of excessive snacking [69]. When you add snacks, blood sugar levels rise and remain high for a long time, which is not a desirable situation in the prevention of diabetes and NAFLD. In addition, peanut butter has the amino acid tryptophan, which helps improve the quality of sleep, prevents depression or anxiety, helps lose weight, and even improves exercise performance; therefore, peanut butter has become especially common among exercisers and health seekers. 

Moreover, peanut butter contains reasonable amounts of available calcium that contributes to strengthening bones, hair, and nails and prevents osteoporosis, it has folic acid and is recommended for pregnant and lactating women, and it has magnesium and potassium that help muscles recover from training [61].

### 2.4. Caffeine

As we have previously shown, nutritional therapy is important for maintaining the state of satiety [70]. It is carried out in order to degrade glucose, fatty acids, and cholesterol, which influence the metabolism of toxic amyloid beta oligomers [71] with great importance in chronic diseases. Caffeine is used as an appetite suppressant [72], but with age, its delayed metabolism can be involved in triggering NAFLD and type 3 diabetes [73]. Daily drinking of at least one cup of coffee is a common habit for more and more people, which is why consumption has increased exponentially in the recent times [74]. In contrast to previous studies, Hayat et al., 2021, showed that regular and moderate coffee consumption in the healthy population is associated with a low risk of NAFLD. In addition, in patients with NAFLD, it reduces the risk of developing fibrosis [75]. This was evident by stimulating the apoptosis of hepatic stellate cells and the expression of cAMP, the suppression of actin synthesis, along with the inhibition of alpha-smooth muscle actin. Moreover, the expression of procollagen was also suppressed [76]. However, it was difficult to identify the source of caffeine responsible for these effects. Interestingly, of all the caffeinated beverages on the market such as energy drinks, expresso, tea, and soda, only moderate and frequent consumption of normal coffee has been shown to be associated with a significant decrease in fibrosis. In addition, it caused an improvement in liver function by decreasing the activity of marker enzymes such as ALT and GGT [77]. 

NAFLD in patients with type 2 diabetes is more frequent and severe, and the risk of cirrhosis and liver cancer is much higher. Coffee is a drink composed of several compounds, the main ones being caffeine and chlorogenic acid. Mansour et al. attempted to demonstrate the effects of chronic administration (6 months) of chlorogenic acid and/or caffeine in patients with type 2 diabetes who also presented NAFLD. After 6 months, no improvement in the activity of the liver markers ALT and GGT was observed [78]. Additionally, the supplementation of the two compounds in the modulation of insulin resistance—homeostasis model assessment–estimated insulin resistance (HOMA–IR)—had no effects. The exception was the decrease in cholesterol after caffeine administration and the increase in insulin in the group that received chlorogenic acid plus caffeine.

In the end, they concluded that the administration of 200 mg/day of chlorogenic acid and caffeine in patients with NAFLD did not significantly change the inflammatory, biochemical, and metabolic parameters [78]. 

MacKenzie et al. reported that caffeine in young adults can decrease insulin sensitivity (400 mg) [79]. Several mechanisms underlying this metabolic change have been suggested, not all of which are fully understood. On the one hand, caffeine inhibits the sensitivity of skeletal muscle for glucose uptake by competitively blocking adenosine receptors. Moreover, glycogen synthase activity is also inhibited [80]. On the other hand, these effects are attributed to the increased concentration of epinephrine and fatty acids that can increase insulin resistance after coffee consumption [80]. However, it remains to be understood whether the positive effects of coffee are the result of other ingredients that annihilate the effects of caffeine on insulin resistance [81]. In rats, it has been observed that the chlorogenic acid from coffee is responsible for lowering glucose concentration. In addition, insulin sensitivity is increased by quinides, which are metabolites of chlorogenic acid [82]. It competitively inhibits glucose absorption in enterocytes. At the same time, by suppressing glucose-6-phosphatase activity, it reduces the synthesis of glucose in the liver [82]. In summary, caffeine from regular coffee may be useful in fibrosis.

## 3. Sweeteners

### 3.1. Stevia

Stevia is a natural sweetener extracted from the ***Stevia rebaudiana*** plant [83]. This plant has been grown for its sweetness and medicinal purposes for centuries in South America. The plant compounds that provide sweetness are known as steviol glycosides. ***Rebaudioside A*** is a glycoside 200 times sweeter than sugar [84]. Of all the steviol glycosides in the plant, ***Rebaudioside A*** has the least bitterness.

The consumption of sugar-sweetened beverages is a greater risk factor for the evolution of non-alcoholic steatohepatitis (NASH). Natural sweeteners such as stevia are food additives that provide sweetness without calories and are considered safe and/or not metabolized by the liver [85]. The potential role of sweeteners such as sugar-sweetened beverages are now known to have serious implications for human health. As a result, non-caloric sweeteners such as aspartame, sucralose, saccharin, and ***Rebaudioside A*** have gained in popularity and use [86]. ***Rebaudioside A*** is an extract of the stevia leaf and provides sweetness without calories. It is worth noting that the literature has shown that ***Rebaudioside A*** may in fact play an important role in glucose metabolism and has even been described to improve the postprandial glucose-insulin index [87], and its consumption may result in weight loss (Table 1).

The effect of stevia consumption in the development of NASH began to be clarified recently in a groundbreaking study [88]. The study aims to determine the effect of sweeteners such as ***Rebaudioside A*** from stevia and sucralose on NASH using a murine model of NASH and obesity by a high-fat diet and by replacing fructose and sucrose with the aforementioned sweeteners in the drinking water [88]. The authors found that sweeteners had no effect on weight increase and energy balance in high-fat diet-induced obesity. However, compared to fructose and sucrose, ***Rebaudioside A*** significantly improved liver enzymes, liver steatosis, and liver fibrosis. In addition, ***Rebaudioside A*** induced enhanced gene expressions related to oxidative stress, improved fasting glucose levels, improved insulin sensitivity and caused an increased pancreatic beta cell mass, as well as caused changes in the composition of the microbiome. The findings allowed for the researchers to conclude that ***Rebaudioside A*** significantly improved the pathological expression of NASH in mice.

The known relationship between nutrition, human health, and gut microbiota is related to the fact that the microbiome is in tight connection with metabolism and immunity. Additionally, it participates in the development of NASH pathology [89]. The composition and function of the microbiome is rapidly modulated by nutrition, such as the fermentation of undigested carbohydrates [90]. It was recently published that ***Akkermansia muciniphila*** bacteria partially counteract obesity and related metabolic diseases [91]. The transplantation of fecal contents from saccharin-fed mice into germ-free mice has been reported to transfer the glucose tolerance phenotype to the recipient mice. In this way, the potential role of microbiome metabolic changes secondary to dietary sweetener consumption was shown [92]. Since ***Rebaudioside A*** is not absorbed in the gut [93], the role of the microbiome may be more significant to define the enhanced metabolic outcomes observed in the previous study [88].

The reported data [88] denote that substituting fructose and sucrose with Rebaudioside A as a sweetener may ensure liver protection. They report that the utilization of Rebaudioside A is related to improved glucose tolerance, lower liver fibrosis, and inflammation mediation through lowered oxidative stress (Table 1). For example, ***Rebaudioside A*** may have the potential to inhibit hepatic oxidative stress and NFκβ-mediated inflammatory response by upregulating the nuclear factor Nrf2. It was also reported [94] that ***Rebaudioside A*** protected human hepatocytes in HepG2 cell culture against carbon tetrachloride-induced oxidative stress and inhibited the development of fibrosis. Specifically, ***Bacteroides*** have been reported to efficiently hydrolyze ***Rebaudioside A*** to steviol [95]. An inverse relationship with ***Akkermansia*** abundance and body weight of mice and humans has also been reported [96]. The study by Xi et al. [88] initially found that the ratio between ***Akkermansia*** and ***Bacteroides*** was improved by ***Rebaudioside A*** compared to sucralose administration.

It can be concluded that stevia has many beneficial effects on the non-alcoholic fatty liver disease (NAFLD) of diabetic rats. Its effectiveness is mainly due to a decrease in oxidative stress and a hypoglycemic effect on the microbiome.

### 3.2. Sucralose and Saccharin

Compared to the above publications, there are several studies that report that the consumption of several sweeteners may disrupt the diversity of the microbiome in both rats and humans [97,98]. The aforementioned studies suggest that this may lead to glucose intolerance. At the same time, additional studies reported that the consumption of sweeteners has no effect on the abundance of the microbiome and gene function, but the consumption of several sweeteners changes the diversity of the microbiome [99]. Additional studies also showed that a change in microbial diversity may lead to metabolic changes [100]. These artificial sweeteners, as mentioned, have been reported to be associated with dysbiosis (Table 1). Dysbiosis, by definition, is “an imbalance in the bacterial community in the intestines associated with diseases” [101]. According to this definition, it can be safely said that, despite previous misconceptions, some sweeteners “unequivocally and irrefutably” disrupt the gut microbiota [95]. Nevertheless, different sweetener formulations may have different effects. Moreover, there are several questions about the extent and nature of what happens after consuming certain sweeteners.

Sucralose is one of the most widely consumed sweeteners worldwide. It is 600 times sweeter than sucrose. Studies indicate that sucralose may cause dysbiosis by reducing the total number of aerobic and anaerobic species, ***Bifidobacteria, Lactobacilli, Bacteriodes,*** and ***Clostridiales*** [102]. Another study showed that it can increase Clostridium XIVa strains in mice [103]. Saccharin, also one of the most common sweeteners in the world, has been studied for its possible role in dysbiosis. Recent data indicate that saccharin may inhibit the growth of six bacterial strains: three species of lactobacilli and three strains of E. coli [95] (Table 1). Another study found that saccharin increases the genus ***Bacteriodes*** and, similar to the previous study, decreases the number of lactobacilli [98]. An important issue that can be emphasized is that most of the available information is based on animals. There is a significant need to examine this possible relationship in human subjects with different dietary approaches due to the fact that, in humans, there are many factors that affect the gut microbiota, and the most important is the diet pattern. In a recent study, it was suggested that, by following a large group of human subjects, the researchers were able to find associations between the consumption of sweeteners and a disturbed microbiota [98]. In summary, the use of sucralose and saccharin sweeteners is not recommended.

### 3.3. Maltitol

Maltitol is a polyol produced by the hydrogenation of maltose [104]. It has a low caloric value and a low glycemic index [104]. Moreover, maltitol has sweetening and satiety effects equivalent to sugar and is currently used as a sugar substitute by diabetic patients.

The cholesterol levels may be suppressed by many indigestible components such as dietary fiber and beta-glucan, through the absorption in the intestine of bile acids [105]. Propionic acid produced by the intestinal bacterial flora, in addition to indigestion, may inhibit cholesterol synthesis in the liver, determining a decrease in blood cholesterol concentration [106,107] (Table 1). Additional studies demonstrate that indigestible fibers as well as soluble fibers, such as maltitol, absorb bile acids in the intestine and reduce circulating bile acid levels, which leads to the activation of bile receptors in the liver and an increase in circulating bile acid levels [108] (Table 1).

A study by Urushima et al. [109] demonstrated that supplementation with maltitol suppressed obesity, hyperglycemia, hypercholesterolemia, and fatty liver degeneration in mice fed a high-fat diet. Therefore, maltitol may be useful for treating patients in the initial stages of fatty liver disease to improve steatohepatitis. They demonstrated that maltitol ameliorates non-alcoholic fatty liver disease by activating the Nrf2 antioxidant capacity.

### 3.4. Erythritol

Erythritol (Ery) is a natural polyol sweetener derived from corn, wheat, and other starches. It has an extremely low energy value and a variety of biological functions. Erythritol contains 0.2 calories per gram and is about 60–80% sweeter compared to sugar. Studies have found that the long-term administration of Ery has no effect on the body weight and glucose tolerance of young/adolescent mice [110]. Another study found that Ery can alleviate metabolic disorders in mice induced by a high-fat diet (HFD), including dyslipidemia, impaired glucose tolerance, and obesity [111]. Ery also has an effect of reducing oxidative stress in diabetic rats [112]. It has been shown that erythritol can effectively inhibit hepatic lipid accumulation and alleviate hepatic oxidative damage in HepG2 cells induced by fatty acid treatment and in high-fat diet-induced NAFLD models [113] (Table 1). The potential mechanism of its protective effect is that erythritol exerts an antioxidant function by activating the Nrf2 signaling pathway, thus inhibiting endoplasmic reticulum stress and lipid accumulation and then playing a role in alleviating NAFLD (Table 1).

Erythritol is absorbed relatively rapidly in the small intestine. Due to the fact that it does not stay in the intestines for long, it cannot attract water—the main cause of watery diarrhea from sweeteners such as maltitol. This is why erythritol causes diarrhea less frequently than other sweeteners such as xylitol [114].

In 64 healthy young adults, erythritol caused fewer digestive problems (bloating, gas) even at a high dose (50 g), compared to xylitol [115]. Therefore, the maximum intake of erythritol is 0.7 to 1 g per kilogram of body weight. In summary, some sweeteners can be used as an adequate alternative replacement for sugar.

## 4. Glutathione and NAFLD

Glutathione, γ-L-glutamyl-L-cysteinyl-glycine, is a tripeptide present in every cell in the human body [116]. Although its roles are complex and remain the subject of ongoing research, glutathione is thought to play crucial roles in cellular detoxification and antioxidant systems, due to the fact that a reduction in glutathione levels in cells has been found to increase the risks of disease and poisoning. Accordingly, direct intravenous injection of glutathione has been used to treat patients with chronic liver diseases and poisonings [117].

Glutathione is formed in cells from glutamic acid, cysteine, and glycine. Cysteine and glycine are formed from methionine and serine, respectively, and glutamic acid is synthesized from α-ketoglutarate, a metabolite of glucose. These amino acids are usually supplied from food. It was reported that oral administration of glutathione did not change blood glutathione and glutathione disulfide levels [118]. It has been suggested that, when glutathione is administered orally, it breaks down into amino acid components and does not exert specific activity beyond the amino acid source. However, it has been reported that glutathione can pass through the Caco-2 cell layer (in vitro system) without degradation [119]. In addition, Park et al. demonstrated an increase in the protein-attached form of glutathione in human blood after oral consumption [120]. These studies indicate that glutathione administered orally is absorbed into the blood and can influence the redox state in the human body.

Glutathione has a long history of treating chronic liver disease by injecting it into a vein. A recent study [121] demonstrated the therapeutic effect of oral glutathione in patients with NAFLD. The main result of this study was a change in ALT levels. The patients with oral treatment of glutathione (300 mg per day) for 16 weeks exhibited a drop in ALT levels as well as low in triglycerides, NEFA, and ferritin levels (Table 2). 

The findings of this study reveal the beneficial effects of glutathione administered through the mouth for NAFLD patients. As we mentioned before, the explanation for this is that glutathione is broken down into amino acids during digestion and absorption processes. The claim today is that, by intaking glutathione orally, it can be used as a source of amino acids in the synthesis of endogenous glutathione. The addition of large doses of glycine and serine (components of glutathione), can also attenuate the severity of NAFLD in humans [121]. In the above study, since the glutathione dose was 300 mg per day, the amount of cysteine potentially released from 300 mg glutathione is less than 120 mg, an amount which can be obtained from between 10 and 20 g of meat or 100 mL of milk. Therefore, it is highly unlikely that the above dose of oral glutathione attenuates the pathogenesis of NAFLD through an amino acid source for glutathione synthesis.

It has been indicated that the level of glutathione in its protein-bound form increases 1–2 h after glutathione ingestion, demonstrating that orally administered glutathione is absorbed into the blood [120]. This protein-bound glutathione may be transported to the liver, thus weakening the hepatitis.

Protein-bound glutathione levels have been reported to return to baseline levels after an overnight fast [122]. In a study by Park et al. [122], they found that the baseline level of the protein-bound form of glutathione decreased significantly after an overnight fast after 16 weeks of glutathione administration. Protein-bound glutathione levels in patients were significantly higher than those of healthy volunteers in previous studies [122] assessed using the same method. Glutathione treatment also lowered protein-bound glutathione to normal basal levels. These findings indicate that oral administration of glutathione may increase the incorporation of protein-bound glutathione in the liver or reduce the pathological secretion of glutathione from the liver.

NAFLD is a complex disease. Its pathogenesis involves various factors, including lipotoxicity, insulin resistance, gut/nutrient-derived signals, oxidative stress, adipocytokines, and genetic factors. In NAFLD, about 20–80% of patients reported dyslipidemia [123]. A previous study indicated that the administration of glutathione accelerates fatty acid utilization by increasing levels of the γ-activated receptor PPR-1α and mitochondrial DNA with reduced levels of nonesterified fatty acids in plasma [124].

An increase in body ferritin and iron stores has been frequently found in NAFLD patients [125]. Ferritin and iron can promote the development of NAFLD through oxidative stress [126]. Results from an experiment conducted on various populations showed that oral administration of the antioxidant Vitamin E improved liver dysfunction and the pathological conditions of NASH [127]. However, long-term treatment with Vitamin E has been associated with increased all-cause mortality and prostate cancer risk [128], suggesting the need to evaluate the efficacy and safety of this agent. In the aforementioned study [127], treatment with glutathione significantly decreased ferritin levels, but the mechanism behind the decrease remains unclear. Glutathione suppresses hyperferritinemia and oxidative stress and has therapeutic effects in patients with NAFLD. Liver fat assessed noninvasively showed that it tended to decrease in all patients and decreased significantly in responders in the decrease of the ALT enzyme after 4 months of glutathione treatment. However, the relationship between histological improvement of liver steatosis and reduction in liver fat values has not yet been determined, including whether glutathione administration may reduce liver steatosis.

Additionally, in the aforementioned work [127], they investigated other factors that could be related to the adequate effects of glutathione. They found that HDL cholesterol and LDL cholesterol levels were higher and that HbA1c levels were lower in patients who responded with a decrease in the ALT enzyme than in those who did not react (Table 2). In summary: glutathione has a therapeutic effect of oral glutathione in NAFLD patients.

## 5. Whole Milk or Low-Fat Milk for Fatty Liver

Milk is an important part of the diet of most people. According to the recommendations, there is no difference in choosing whole milk or low-fat milk if you drink up to one glass of milk per day; it should not pose a significant problem. The benefit of milk in fatty liver is reflected in the consumption of the protein found in milk. Milk protein consumption has been shown to be inversely related to the development of NAFLD [129].

Several possible mechanisms may explain the association of increased protein intake from milk with reduced risk of incident NAFLD. First, mitochondria are considered essential for the development of NAFLD [130]. Reduced β-oxidation, together with increased lipogenesis, production of reactive oxygen free radicals, and damage to hepatocytes lead to lipid accumulation as well as inflammation and fibrosis in hepatocytes [130]. Increased production of oxidative radicals and depletion of antioxidants such as glutathione, Vitamin E, β-carotene, or Vitamin C in the liver may occur in NASH [131]. In a study conducted on rats [132], a significant increase in glutathione was observed only when rats were fed whey protein hydrolysates and β-lactoglobulin. A higher intake of milk protein may (1) help suppress NAFLD synergistically with exercise, and (2) prevent sarcopenia, a known risk factor for NAFLD [133]. Insulin resistance is a key factor in the development of NAFLD. A prospective study in a population of young adults found an inverse relationship between the frequency of consumption of dairy products and the development of insulin resistance syndrome [134].

## 6. Soluble Dietary Fiber (FDS)

Many recently published studies have revealed that bacterial flora and dietary microbial metabolites, such as short-chain fatty acids (SCFAs), contribute to homeostasis as well as to the prevention of the development and progression of various diseases in humans including NAFLD and NASH [135]. Recently, an imbalance in the microbiota, in the bacterial flora of the digestive system, known as dysbiosis, has been indicated, and is involved in a variety of metabolic diseases, including fatty liver [136].

In NASH, dysbiosis can result from an unbalanced diet or obesity, and a reduction in SCFA production can lead to a dysfunctional intestinal mucosal barrier and immunological disturbances [137]. Studies have shown that a large number of pathogen-related molecules can reach the liver through the disrupted intestinal mucosal epithelial barrier, causing the hypersensitivity of Kupffer cells and potentially leading to NAFLD and NASH [138]. 

A recently published study attempted to explain the mechanism underlying the possible amelioration caused by the administration of fructo-oligosaccharide (FOS) in improving NASH disease in mice. They reported that increased SCFA production by the bacteria provided nutrients to intestinal epithelial cells, thus improving intestinal barrier function. The effect was also to increase immunoglobulin A production and suppress Kupffer cell activation in NASH-induced mice [139].

There are three other potential explanations for the observed beneficial effects of SCFA in the study in NASH. First, SCFAs stimulate the secretion of peptide-1 (GLP-1) from the L cells in the gastrointestinal tract [140], given that several recent clinical studies have demonstrated that treatment with GLP-1 can regulate the accumulation of lipids in the liver [141]. Additional studies have shown the effectiveness of GLP-1 in the treatment of NAFLD [142]. Second, adipocytes express G-type receptor (GPR43) SCFAs [143]. It was recently reported that GPR43 can act to suppress insulin signaling in adipocytes and inhibit fat accumulation in adipose tissue as well as promote lipid and glucose metabolism in the liver [144]. Third, SCFAs can act as potential ligands for the peroxisome proliferator-activated receptor γ- (PPARγ), and as a result can result in improved insulin sensitivity (Table 1). It was also reported that a significant reduction in the improvement of steatosis was caused by the administration of SCFAs in the liver in mice lacking PPARγ in the liver [145]. It was also reported that the addition of butyrate to the diet caused a decrease in pro-inflammatory markers such as interleukin-6 and nuclear factor-kappa-beta (NF-κB), thereby raising the threshold for inflammatory reactions in the liver of rats fed a high-fat diet [146] (Table 1).

**Table 1:** The influence of bioactive aliment compounds on NAFLD.

Nutrients/Category	Effects on Liver	Effects on Intestinal Microbiota	References
Dark chocolate	1. Positive effects on the lipid profile, reducing total and LDL cholesterol levels and increasing HDL levels		[36]
	2. Improve insulin resistance through reducing oxidative stress, improving endothelial function, and/or altering glucose metabolism		[39]
	3. Decrease aspartate aminotransferase (AST) levels in the serum of NAFLD patients		[44]
	4. Increase glucose uptake, increase fatty acid and glucose oxidation, inhibit lipid synthesis		[45]
	5. Anti-inflammatory properties, which can regulate the TNF-κB gene expression and reduce inflammatory biomarkers and ROS production		[50]
Cocoa butter	1. Lowers cholesterol LDL levels and increases cholesterol HDL levels		[56]
Peanut butter	1. Lowers cholesterol LDL levels and increases protective cholesterol HDL levels.		[67]
	2. Prevents cell damage and induces cell repair, effects associated with reduced risk of chronic diseases such as NAFLD		[59,61]
Caffeine	1. Lowers the risk of NAFLD in healthy people		[75,77]
	2. Reduces the risk of developing fibrosis		
Sweeteners			
Stevia	1. Plays a role in glucose metabolism and has even been reported to improve the postprandial glucose–insulin index		[87]
	2. Significant improvement of liver enzymes blood levels, improvement of liver steatosis and liver fibrosis	1. Causes changes in the composition of the microbiome.	[88]
	3. Decreased inflammation associated with oxidative stress	2. An inverse relationship with Akkermansia abundance associated with body weight of mice and humans	[96]
	4. Lower gene expression related to oxidative stress. Improve fasting glucose levels and improve insulin sensitivity		
Sucralose and saccharin		1. Sucralose may cause dysbiosis by reducing the total number of aerobic and anaerobic species, bifidobacteria, lactobacilli, Bacteriodes, and Clostridiales	[102]
		2. Saccharin may inhibit the growth of six bacterial strains: three species of lactobacilli and three strains of *E. coli* in animal models	[95]
		3. Associations between the consumption of sweeteners and a disturbed microbiota	[98]
Maltitol	1.Suppress cholesterol synthesis in the liver leading to a decrease in circulating cholesterol levels		[106,107]
	2. Maltitol mimics indigestible fibers, absorbs bile acids in the intestine, and reduces circulating bile acid levels, which leads to the activation of bile receptors in the liver and an increase in circulating bile acid levels		[108]
	3. Prevents obesity, hyperglycemia, hypercholesterolemia, and fatty liver degeneration in mice fed a high-fat diet		[109]
Erythritol	1. Long-term administration of Ery has no effect on body weight and glucose tolerance of young/adolescent mice		[110]
	2. Alleviate metabolic disorders in mice induced by a high-fat diet (HFD), including dyslipidemia, impaired glucose tolerance, and obesity		[111]
	3. Inhibits hepatic lipid accumulation and alleviate hepatic oxidative damage in HepG2 cells induced by fatty acid treatment and in high-fat diet-induced NAFLD models		[113]
	4. Exerts an antioxidant function by activating the Nrf2 signaling pathway, thus inhibiting endoplasmic reticulum stress and lipid accumulation and then playing a role in alleviating NAFLD.		
Soluble dietary fiber (FDS)	1.Improvement caused by the administration of fructo-oligosaccharide (FOS) in improving NASH disease in mice	1. Improvement of intestinal barrier function	[139]
	2. Regulate the accumulation of lipids in the liver		[141]
	3. Inhibit fat accumulation in adipose tissue as well as promote lipid and glucose metabolism in the liver		[144]
	4. Provide potential ligands for the peroxisome proliferator-activated receptor γ- (PPARγ), and as a result can result in improved insulin sensitivity		[145]
	5. Decrease expression of pro-inflammatory markers such as interleukin-6 and nuclear factor-kappa-beta (NF-κB), thereby raising the threshold for inflammatory reactions in the liver of rats fed a high-fat diet		[146]

LDL, Low-density lipoprotein; HDL, High-density lipoprotein; VLDL, Very low-density lipoprotein; TNF-α, Tumor necrosis alpha; NF-κB, Nuclear Factor-Kappa beta; ROS, Reactive oxygen species; AST, Aspartate aminotransferase; ALT, Alanine transaminase; NASH, Non-alcoholic steatohepatitis; NAFLD, Non-alcoholic fatty liver disease; TGs, Triglycerides; MDA, Malondialdehyde; IL-1β, Interleukin-1 beta; IL-6, Interleukin-6.

A current study found that FDS significantly increased the concentration of propionic acid in the serum of NASH-induced mice and decreased the mRNA expression levels of the rate-determining enzyme for glycerolipid-glycerol-3-phosphate acyltransferase [147]. It has been reported that propionic acid decreases the hepatic mRNA and protein expression of lipid biosynthetic enzymes [139,148], increases the expression of glucose transporter type 4 (GLUT4), improves insulin sensitivity [149], and inhibits lipopolysaccharide (LPS)-stimulated TNF α release by neutrophils [150]. In summary, FDS inhibits fat accumulation in adipose tissue as well as promotes lipid and glucose metabolism in the liver.

## 7. Soy Lecithin as a Source for Choline and Inositol

Lecithin in the context of NAFLD is considered one of the most important sources of choline and inositol. There is a general lack of awareness about the importance of the essential nutrient choline. This is reflected in the consumption of less than recommended levels of choline by most people. In fact, this relatively low intake of choline by the general population may be directly linked to the great incidence of NAFLD in the Western world, as choline deficiency is known to cause “fatty liver” or steatosis in animals and therefore similarly in humans. Despite the current reported deficiencies, the importance of the nutrient choline has long been demonstrated [151]. Despite the possibility that choline can be obtained from its endogenous biosynthesis, the American Institute of Medicine recognized its nutritional importance and established the minimum values for choline already in 1998.

It is well known that optimal nutrition is extremely important in reducing the risk of metabolic disorders. Maintaining liver health has also been linked to adequate intake of certain nutrients; one of the most important is choline. Choline and phosphatidylcholine (PC), and therefore lecithin, are known to prevent the development of fatty liver [152]. There is now consensus that the potential for increased consumption of choline and lecithin may prevent metabolic pathologies of the liver and other parts of the body [153].

Dietary choline deficiency has multiple consequences for human health, including birth defects, neurological dysfunction, and the development of fatty liver [154]. With regard to steatosis, or fatty liver, choline deficiency has been shown to play an important role. Phosphatidylcholine (PC), found in lecithin, as mentioned above, is an essential structural component of VLDLs and is required for its secretion and the export of triglycerides (TGs) from the liver [155]. Therefore, choline and subsequent PC deficiency may cause fatty accumulation in the liver. In addition, the roles of betaine in homocysteine methylation, antioxidant activity, and AMP-activated protein kinase (AMPK) stimulation have been investigated in relation to NAFLD. In fact, choline and betaine have been shown in animal and human studies to prevent and even ameliorate NAFLD [155] (Table 2).

Currently, only low quantities of lecithin are used in processed foods, usually due to its role as an emulsifier. Increasing the lecithin content in foods for the purpose of improving human health may definitely be an advantage since many people rely on processed foods and do not consume natural sources of choline. A functional food enriched with lecithin will definitely benefit foods that are natural sources of choline, such as soy products, milk, and peanut butter [156]. This may also be an effective strategy in light of the research showing that the consumption of lecithin by humans effectively increases choline levels [157]. Increased levels of lecithin may play a direct role in the prevention of NAFLD based on the essential role of PC as an essential phospholipid in ensuring adequate TG export from the liver (Table 2). Thus, with increased lecithin intake, the incidence of NAFLD can be reduced in the general population. However, the success of functional food depends, at least in part, on consumer awareness of the aforementioned nutrient.

In summary, choline, phosphatidylcholine, and lecithin are associated with preventing the development of fatty liver.

## 8. Turmeric and Curcumin Extracts

Turmeric *(Curcuma Longa)* has active ingredients called curcuminoids with the most prominent curcuminoid being called curcumin. In vitro and animal studies, turmeric has demonstrated potent antioxidant, anti-inflammatory, and anti-fibrotic properties, as well as insulin-sensitizing effects [158]. As such, it may be promising in the treatment of patients with NAFLD.

Several controlled studies investigated the effects of curcumin consumption on anthropometric measures including BMI and body weight and in patients suffering from NAFLD [159,160]. The essential findings of these studies showed that curcumin supplementation (a dose ranging from 80 to 1500 mg per day) significantly dropped BMI and waist circumference in NAFLD patients [159,160].

Even though curcumin has a very low water solubility, it represents an option for use as a dietary supplement and drug [161]. Therefore, its effectiveness on health variables in randomized controlled trials is limited [162,163]. In recent years, researchers have attempted to develop a more water-soluble and more available form of curcumin such as an amorphous form, phospholipid complexes, the addition of piperine (black pepper), liposomal curcumin, and nanoparticles [164]. The nonlinear dose–response test showed the significant effects of nanocurcumin (80 and 400 mg/day) on abdominal obesity, while the natural forms of curcumin (1000 and 1500 mg/day) had less effect on this parameter [159].

Several mechanisms have been proposed for the effects of anti-inflammatory diets and their components, such as curcumin on obesity, and this includes the inhibition of lipogenesis and inflammation (reduction in pro-inflammatory cytokines), the suppression of angiogenesis in adipose tissue, the reduction in preadipocyte differentiation, the increase in lipolysis, and the activation of brown fat. Additionally, the increased energy metabolism of adipocytes and/or the induction of apoptosis increased the expression of neseptin levels in serum and probiotic-like effects [165,166,167]. The effects of nesceptin include loss of appetite, reduction in body fat, anti-inflammatory activities, anti-hyperglycemic activity, as well as metabolic and neuroendocrine regulation [168,169]. Thus, the improvements in NAFLD, anthropometric indices, inflammation, glucose, and lipid metabolism, and subsequently the increased levels of neseptin by curcumin supplementation can be noted. Additionally, curcumin may reduce total body fat by increasing the basal metabolic rate [170].

The enzyme 1β-hydroxysteroid dehydrogenase 1 is expressed in adipose tissue and the liver. This enzyme can increase the cortisol level in visceral (abdominal) fat by changing the inactive form of the cortisol hormone to its active form [171]. A high amount of cortisol hormone in adipocytes can cause adipogenesis and consequently central obesity [172]. Therefore, the inhibition of the enzyme 11β-hydroxysteroid dehydrogenase 1 can be effective for reducing visceral fat and treating the metabolic syndrome. According to a previous study, curcumin can act as an inhibitor of this enzyme [173].

Curcumin reduces body fat mass by inhibiting adipocyte differentiation through the suppression of peroxisome proliferator-activated receptor-γ and by increasing adenosine monophosphate-activated protein kinase, resulting in lipolysis [174]. From all of the above, it can be assumed that curcumin supplementation (especially nano-curcumin) may have a moderate effect on BMI in patients with NAFLD.

The current therapeutic strategies for the treatment of NAFLD and NASH are mainly aimed at correcting and changing risk factors such as obesity, diabetes, and hyperlipidemia. However, there are several studies that indicate that curcumin can also intervene in the oxidative stress that occurs in NAFLD [160]. Additionally, the turmeric plant (Curcuma longa) or its component is indeed likely able to protect the liver with an anti-oxidant mechanism; therefore, it has been used not only as a spice but also as a traditional medicine for many centuries, and its properties have been reported in the literature [175]. A clinical trial was recently conducted to evaluate the potential role of orally administered turmeric on liver enzymes, lipid profile, oxidative stress status, malonaldehyde (MDA), and degree of hepatic steatosis [176]. The study included 62 patients who were randomly divided into intervention groups or placebo (wheat flour). The participants in the intervention group received a turmeric supplement (2 g per day) as oral capsules and the other group received a placebo. The intervention period was 8 weeks and the subjects were advised to consume their capsules after the main meals to improve absorption in the small intestine due to the presence of fat in the diet. The study revealed that taking a supplement containing 2 g per day of turmeric for a period of 8 weeks resulted in a significant decrease in the degree of steatosis compared to the beginning as seen in the liver ultrasound tests. The results of the clinical study showed that supplementation with turmeric extracts reduces elevated serum ALT and AST levels in patients with NAFLD (Table 2). A decrease in these two enzymes can indicate an improvement in liver function. Therefore, it can be considered as an adequate therapeutic supplement with hypolipidemic and antioxidant properties for this disease. However, the reports in other studies do not always support the above results.

Previous studies have also highlighted the mechanism of the hepatoprotective effect of curcumin [177] in both in vitro and in vivo studies. The antioxidant capacity of curcumin in scavenging free oxygen radicals, reactive nitrogen molecules, and lipid radicals is one of the most important mechanisms [178]. The role of oxidative stress and inflammation in inducing hepatocyte injury and progression of NAFLD has long been established in previous studies [179]. Curcumin treatment also enhances the activities of detoxifying enzymes such as glutathione-S-transferase, glutathione peroxidase, glutathione reductase, catalase, and he-oxygenase-1, and thus suppresses oxidative stress in the liver [179,180,181]. Curcumin inhibits the activation of key mediators of cellular inflammation such as 5-lipoxygenase (5-LOX), NF-κB, and cyclooxygenase-2 (COX-2). These are involved in the stimulation of different genes including several pro-inflammatory and cytotoxic cytokines such as TNF-α, IL-1, IFN-γ, and NF-KB [182,183]. Curcumin also suppresses the activation and proliferation of stellate cells in the liver, which have a known role in the progression of liver fibrosis [184] (Table 2). A decrease in hydroxyproline content in the liver and a downregulation of collagen mRNA synthesis after curcumin administration supports this claim [185].

## 9. Silymarin

Silymarin is derived from an extract from milk thistle (*Silybum marianum* L.), a plant that originates from southern Europe through Asia with the active compound silymarin, which is a natural flavonoid. Silymarin consists of four flavonolignan isomers, namely silybin, silychristin, silydianin, and isolibin [186], and it is widely used as an over-the-counter preparation for liver diseases [187]. Its beneficial effect on the liver is attributed to possible anti-inflammatory, antioxidant, and anti-fibrotic activity. Silymarin also reduces insulin resistance [188]. It has been tested in the treatment of various liver diseases, exhibiting adequate results with remarkable safety [189].

Today, it is the most studied plant for the treatment of liver diseases and the most common over-the-counter treatment for liver diseases [190]. Several studies have indicated its promising antifibrotic activity in liver injury in experimental systems [191,192] (Table 2). Silymarin may reduce liver inflammation by inhibiting lipooxygenase activity and reducing the function of leukotrienes and their effect on Kupffer cells in the liver and by reducing oxidative stress by increasing glutathione levels [193].

Treatment with milk thistle extract significantly improved serum superoxide dismutase activity and malondaldehyde (MDA) levels in rats in which NAFLD was induced [192]. Milk thistle extract treatment reduced serum aspartate aminotransferase enzyme levels and levels of triglycerides (TGs), as well as cholesterol including VLDL in NAFLD-induced rats (Table 2). Treatment with milk thistle extract preparations also effectively protected the liver against histological changes. From these data, it can be concluded that treatment with milk thistle extract preparations can be a promising medicinal option for the treatment of NAFLD.

Recently, several clinical trials have been conducted to treat NAFLD with milk thistle extract preparations. A recent randomized controlled trial (RCT) [194] concluded that silymarin was effective in reducing alanine aminotransferase (ALT) and aspartate aminotransferase (AST) levels compared to the placebo treatment. In a double-blind, placebo-controlled RCT by Kheong et al. [195] with adults with NASH, it was demonstrated by biopsy that 48-week silymarin treatment did not lead to an improvement in the NAFLD activity score compared to the placebo group, but it did lead to a significant improvement in fibrosis after repeat liver biopsies. Another clinical trial in NAFLD patients, conducted by Solhi et al. [196], tested the effect of silymarin after a treatment period of 8 weeks. They demonstrated that there was a marked improvement in transaminase levels compared to placebo. Besides the mentioned RCTs that tested silymarin monotherapy in NAFLD/NASH versus placebo treatment, other studies with methodological shortcomings or not placebo-controlled were also performed [197,198].

In conclusion, turmeric, curcumin extracts, and silymarin may be therapeutic for NAFLD patients.

## 10. Selenium (Se)

There are animal studies that indicate a link between selenium (Se) supplementation and NAFLD. Although most indicate a beneficial effect, some report a negative effect of Se supplementation on NAFLD.

### 10.1. Evidence Suggesting a Beneficial Effect of Se on NAFLD

Rats fed a Se-deficient diet exhibited a decrease in reduced glutathione (GSH) in the liver and an increase in the ratio of n-6/n-3 fatty acids, which are considered to be detrimental to NAFLD [199]. Additionally, mice fed a Se-deficient diet exhibited an increase in lipid peroxidation and a decrease in glutathione peroxidase (GPx) and thioredoxin reductase activity in the liver, suggesting increased oxidative stress and impaired antioxidant capacity [200], both of which are strongly associated with the pathogenesis of NAFLD [201]. Furthermore, rats fed a Se-deficient diet had lower levels of Se in the liver, which resulted in changes in hepatocyte cells, such as abnormal chromatin and mitochondrial swelling, as well as more severe fibrosis around the portal vein, upregulation of metalloproteinases, and downregulation of tissue inhibitors of metalloproteinases type 1 and 3, all of these changes being associated with increased liver inflammation [202]. 

Liver fibrosis induced by N-Nitrosodimethylamine treatment in rats was associated with decreased circulating Se, decreased hepatic GSH and GPx, and increased circulating IL-6 and TGFβ1 cytokines. Another study demonstrated that low Se levels and consequently lower -GPx activity may impair cellular antioxidant defense, leading to oxidative stress and liver fibrosis [203]. In addition to the link between Se deficiency and NAFLD, there are also studies that indicate a positive effect of Se supplementation, alone or in combination with other drugs, on liver function tests and liver steatosis (Table 3). 

Se supplementation improved abnormal liver functions induced by carbon tetrachloride (CCl4) treatment in rats [204]. The administration of Se plus magnesium prevented high-fat diet (HFD)-induced lipid increase in rats, possibly by enhancing the activity of several antioxidant enzymes [205]. Another study showed that the administration of a combination of Se and zinc improved the lipid profile, liver functions, and liver steatosis in rats [206]. Additionally, the administration of Se and probiotics reversed the negative effect of feeding in mice on HFD and improved liver functions and steatosis [207]. Another study demonstrated that administering selenomethionine (the organic form of selenium which is the same form found naturally in foods such as grains, soybeans, and yeast) to rats induced steatosis. The improvement was accompanied by increasing GPx activity, weakening the steatosis in the liver and allowing for the appearance of hepatocytes in a balloon-like configuration (a hallmark of hepatitis) [208]. The effect of Se supplementation on liver fibrosis, the principal histological prognostic factor for advanced disease observed in the study [208], appears to be even more important. Other studies demonstrated that Se supplementation decreased the number of hepatic stellate cells (HSCs) and liver fibrosis induced by CCl4 treatment in mice [209]. It should be emphasized that HSCs are considered to be key players in the pathogenesis of liver fibrosis [210] (Table 3).

### 10.2. Evidence Suggesting a Negative Effect of Se Administration on NAFLD

Contrary to the evidence presented above, some articles reported a negative effect of Se administration on NAFLD, which, at the same time, may be related to exposure to high doses of Se (higher doses and/or longer duration). Early reports suggested that oral administration of Se for 2 months in rats induced the formation of nodular regenerative hyperplasia with sinusoidal damage in certain areas of the liver [211]. These areas are found around nodules (perinodular areas) and in which atrophic hepatocytes appear around capillary sinusoids but without fibrosis [212]. Based on these findings, another study showed that a Se-enriched diet induced hepatic nodular hyperplasia in rats [211].

## 11. The Enzyme Stearoyl-CoA Desaturase 1 (SCD1) in NAFLD and the Use of Supplements That Lower Its Activity

In many tissues, stearoyl-CoA desaturase 1 (SCD1) catalyzes the biosynthesis of monounsaturated fatty acids (MUFAs), (i.e., palmitoleate and oleate) derived from their saturated fatty acid (SFA) counterparts (i.e., palmitate and stearate), leading to broad effects in terms of physiology in humans. In addition to its main role in fatties metabolism and body weight control, SCD1 has recently appeared as a potential new target for the treatment of various diseases, such as cancer, Alzheimer’s disease, skin disorders and, in our case, NAFLD [213].

Under normal conditions, lipogenesis and lipolysis are in dynamic balance. Signals arrive from both the central nervous system, as well as peripheral tissues, inducing the balance of synthesis and breakdown of triglycerides. There are two different sources of lipogenesis in the endogenous formation of fats (de novo lipogenesis). In tissues with a high metabolic rate, such as the liver or adipose tissue, de novo lipogenesis is more active, although every single cell is capable of performing lipogenesis. In particular, human adipose tissue appears to be the primary tissue in which de novo lipogenesis takes place [214]. This type of lipogenesis is characterized by the conversion of carbohydrates into fatty acids, which are then stored as triglycerides if the body does not need energy. This process begins with the glycolysis of carbohydrates to obtain acetyl-CoA. The enzyme acetyl-CoA carboxylase 1 (ACC1) converts acetyl-CoA into malonyl-CoA, which is then converted to palmitate by fatty acid synthase (FASN) [214]. Finally, the last step of de novo lipogenesis is carried out by stearoyl-CoA desaturase (SCD), the first rate-limiting enzyme involved in desaturation [215]. SCD catalyzes the reaction in converting palmitoyl-CoA to palmitoleoyl-CoA, in a reaction that also involves redox by nicotinamide adenine dinucleotide (NADH), flavoprotein cytochrome β5 reductase, after receiving the electrons from cytochrome β5 [216]. In addition to the palmitic acid reaction, stearic acid is also one of the major substrates of SCD, which is ultimately converted into oleic acid [217]. SCD also catalyzes the conversion of myristic acid into myristolic acid [218] but this reaction is carried out to a lesser extent.

De novo lipogenesis is triggered when blood glucose and insulin levels are increased [214]. These nutrients cause the activation of the transcription factor ChREBP by using another transcription factor, SREBP-1c, and the liver type receptor (X -LXR). These transcription factors are specific and their activation promotes lipogenesis again [215]. Thus, the hepatic expression of SCD-1 is induced after high-carbohydrate consumption through an SREBP-1c-dependent mechanism involving the binding of LXR to the LXR response to the promoter activation of SCD-1 through the transcriptional activation of SREBP-1c [219]. Pharmacologically administered SCD1 inhibitors have been tested with adequate results in NAFLD, diabetes, dyslipidemic failure, and hepatitis C virus infections [220]. For example, a drug called MK-8245 was developed and is currently in advanced clinical trials in humans, including against NAFLD [221].

Researchers have reported that an SCD inhibitor called Daiichi Sankyo [222] may be effective for the treatment of NASH. The Daiichi Sankyo compound was given orally once daily at a dose of 30 or 100 mg per kg to rats fed a methionine-deficient choline diet for 2 months before treatment of the SCD inhibitor. After 1 month of administration with the above compound (100 mg/kg), there was a reduction in the accumulation of triglycerides in the liver of rats suffering from NASH by 80% (Table 4). The Daiichi Sankyo compound also reduced the increase in aspartate aminotransferase (AST) enzyme levels and alanine transaminase (ALT) by 86% and 78%, respectively. Hepatic steatosis, hepatocellular degeneration, and inflammatory cell infiltration were also treated after treatment with this compound (Table 4).

### Adverse Effects of SCD1 Inhibitor Treatments

It is important to note that SCD suppression can also produce unwanted effects in mammals. Tissues, where lipogenic mechanisms have to be active to function normally, can be affected by long-term systemic treatments with SCD1 inhibitors. For example, SCD1 knockout mice develop multiple skin eczemas accompanied by weight disturbances [223]. These animals exhibit dysfunction of the epidermal lipid barrier with subsequent thermoregulation failure, transepidermal water loss, and metabolic problems [224]. The SCD1-deficient mice also suffer from atherosclerosis accompanied by an increased inflammatory response of the macrophages [225]. In addition, treatments with SCD1 inhibitors usually cause the atrophy of sebocyte cells (epithelial cells in the skin), due to which hair loss and dry eyes are caused [226]. On the basis of these findings, the development of new SCD inhibitors with fewer adverse effects, as well as a better understanding of the mechanisms involved, are of crucial interest. Using nutritional supplements that perform the activity in a moderate way is possible. An example is sterculic acid (SA) (see below).

## 12. Sterculic Acid (SA)

Sterculic acid (SA) is a cyclopropane fatty acid with many biological activities. SA is a fatty acid mainly obtained from the seeds of *Stracolia petida* and it forms more than 50% of its oil composition. SA is known for the inhibitory effect it exerts on the SCD1 enzyme, both in vivo and in vitro [227,228]. Studies in adipocyte cells have shown that this inhibition occurs by regulating enzyme activity without affecting SCD mRNA levels or protein expression [229]. Thus, this inhibition could be due to the irreversible binding of the sulfhydryl groups of the enzyme with cyclopropane groups found in sterculic acid [230] or through the conversion of SA to stearoyl-CoA, which appears to be the active form [231].

SA is one of the main components of sterculic oil (SO) [232]. SO contains not only SA, which is its main component, but also malvalic acid, another cyclopropane acid with properties similar to that of SA, including an inhibitory ability of SCD [233]. The beneficial effects of SO include improvement in terms of glucose tolerance and blood pressure, reduction in body mass, and benefit in serum levels of triglycerides and adiponectin [228] (Table 2).

However, a number of side effects have also been described. These include hypercholesterolemia, reproductive problems in animals, and inhibition of the beneficial effect of CLA (conjugated linoleic acid) in rats due to disruption of SCD activity [234].

## 13. Aquamin

Aquamin is a multimineral complex containing calcium, magnesium, and 72 other marine minerals that are absorbed from the surrounding seawater. It can be used in foods, beverages, and nutritional supplements. 

The effect of Aquamin treatment was tested in a murine model of NAFLD. Groups of mice were fed a high-fat diet with fructose added to the drinking water with and without the addition of Aquamin for a period of 16 weeks [235]. They proved that Aquamin, which is rich in calcium, magnesium, and other elements (derived from red algae), prevents and can even help stop the progression of NAFLD (Table 2). Previous studies by this group [236,237], were conducted with black mice that were fed a high-fat Western-style diet (HFWD) for up to 18 months. The long feeding period of HFWD allowed for the development of extensive liver damage in most animals, especially males. In addition to the widespread steatosis found in mice fed HFWD, the mice exhibited widespread inflammation of the liver, liver injury, and the development of collagen deposits (Table 2). The diet caused the appearance of large fibrotic nodules. It was even possible to identify liver tumors including liver adenomas and carcinomas in several animals. Their studies demonstrated that supplying an adequate level of calcium (estimated as 20–25 mg per day consumed) together with several other trace elements in the mineral supplement Aquamin dramatically reduced the formation of tumors [236,237]. Inflammation, damage to hepatocytes, and the appearance of collagen deposits were also reduced, but the steatosis indices themselves were largely unaffected.

Calcium, which is the distinct “driver” of epithelial cell differentiation [238], is the most abundant mineral in Aquamin. Thus, epithelial cell differentiation induced by calcium in the multimineral product may underlie the suppression of the precancerous and cancerous processes in the liver induced by the HFWD diet and prevented by Aquamin treatment. Furthermore, it is well documented that some of the trace elements in the Aquamin multimineral product can act as calcimimetic (calcium-like) agonists, promoting the calcium response. Support for this is that an extracellular calcium-sensing receptor is also expressed in rat hepatocytes [239]. Inadequate mineral intake is not limited to people consuming a Western-style diet. A recent study showed that most people living in many developing regions of the world also lack an adequate amount of calcium in their diet [240]. The question that is asked is whether the mineral supplement Aquamin may ensure the consumption of minerals at an adequate level. A recent 90-day pilot trial was conducted in which 30 healthy subjects were randomly divided into several groups: one group designed to receive Aquamin that should provide 800 mg of calcium per day, another group that received calcium carbonate at the same level, and a third group that received a placebo [241]. In conclusion, no safety or tolerability issues were seen with Aquamin. At the same time, the colon biopsies obtained before and after the treatment demonstrated the regulation of several proteins related to cell differentiation in the colon mucosa. In the calcium-only group, differentiation proteins were also formed, but the levels of increase were much lower compared to what was seen with Aquamin. Finally, a decrease in the levels of certain primary and secondary bile acids was also observed in subjects who received Aquamin in combination with a change in the intestinal bacterial profile. These metabolic and microbial changes were not observed with calcium alone. While the focus of these clinical studies was colon health, the same approach may also provide benefits in terms of liver health.

In conclusion, previous studies have clearly demonstrated the importance of adequate mineral intake to prevent the consequences of fat accumulation in fatty liver in a murine model. Current studies attempt to provide mechanistic insight into how mineral supplementation may contribute to a reduction in liver tumor formation in a murine model, one of the most devastating consequences of fatty liver disease versus steatosis. There is currently no evidence of the effectiveness of using Aquamin to prevent NAFLD in humans.

## 14. Oleic Acid

The main fatty acid provided by olive oil is oleic acid or oleate (C18:1 n-9), and it is known for being the main contributor to the beneficial effects of olive oil consumption (Hu, 2003).

Ducheix et al. [242] recently investigated the effects of dietary-supplied oleic acid on the regulation of gene expression in the liver. Indeed, while oleic acid can be synthesized de novo through the activity of Stearoyl-CoA desaturase 1 (SCD1), it is clear from the results obtained in mice lacking the *SCD1* gene (general *Scd1 KO*) [243] as well as in liver-specific *Scd1 KO* mice [244] and in mice overexpressing *Scd3* [245] that oleate can positively contribute to various physiological functions, mainly in the liver. Dietary-supplied olive oil [246] and oleic acid [247] have been shown to have beneficial effects in various experimental models of liver pathologies in NAFLD, ranging from steatosis to steatohepatitis (NASH). In addition to the effect that prevents the accumulation of lipids in the liver, oleic acid synthesized de novo also contributes to the protection of hepatocytes against insulin resistance [248].

Recently, it has been shown that oleic acid can modulate the activity of liver X receptors (LXRs) in human neutrophils [249] (Table 2). Moreover, LXR activity is sensitive to fatty acids as tested in vitro [250]. The LXRs are type II nuclear hepatocyte receptors [251]. They are sensitive to derivatives of oxidized cholesterol, the oxysterols, which bind to and activate both isotypes of LXR (β α, NR1H3 NR1H2). An increase in the oxysterol concentration stimulates the transcription of the LXR target genes. For example, LXR α and β regulate genes involved in lipogenesis [252]. LXRα binds to transcription factors responsible for the control of lipogenic genes such as *Fasn* (responsible for fatty acid synthesis) [253] or *Scd1* [254] and directly promotes de novo fatty acid synthesis. Therefore, the pharmacological activation of LXR leads to the accumulation of neutral lipids in the liver, which is the hallmark of NAFLD [255]. LXRs are also involved in the reverse transport of cholesterol and the breakdown of cholesterol into bile acids. LXR is not only involved in cholesterol secretion [256] but also in suppressing inflammation [257], and thus may be involved in protecting the liver against inflammatory processes that may occur in NAFLD [258]. Indeed, the contribution of LXR to the modulation of lipogenesis, cholesterol metabolism, and liver inflammation by dietary oleic acid was recently investigated [242]. It was demonstrated that LXR is required for the lipogenic genetic response as well as for the decrease in cholesterol in response to a diet that provides a high content of oleic acid. Moreover, it was identified that, in this process, LXR protects against inflammation and liver damage caused by lipogenesis. The authors’ work reveals that LXR contributes to the effects induced by dietary oleic acid and protects the liver from inflammation while inducing lipogenesis. Support for the above findings was published in the in vivo work carried out by Moravcová et al. [259], who found that in in vitro models of steatosis, oleic acid protects against the cytotoxic activity of steatosis caused by treatment with palmitic acid in primary rat hepatocytes in culture (Table 2).

**Table 2:** General and nutritional-associated compounds with beneficiary action on NAFLD.

Compounds		Effects on Liver	Effects on Intestinal Microbiota	References
Glutathione		1. Therapeutic effect of oral glutathione in patients with NAFLD		[121]
		2. Improvement of ALT blood levels		[122]
		3. Decrease in triglycerides, NEFAs, and ferritin levels		[127]
		4. Lowers protein-bound glutathione to normal basal levels		
		5. Improvement of hyperferritinemia and oxidative stress, and exertion of therapeutic effects in patients with NAFLD		
Soy lecithin as a source of choline and inositol		1. Lecithin in the context of NAFLD is considered one of the most important sources of choline and inositol		[151]
		2. Choline, phosphatidylcholine, and lecithin are associated with the prevention of the development of fatty liver		[152]
		3. Choline and betaine have been shown in animal and human studies to prevent and even ameliorate NAFLD		[155]
		4. Lecithin ensures adequate TG export from the liver		[157]
Turmeric and curcumin extracts		1. Antioxidant, anti-inflammatory, and anti-fibrotic properties, as well as insulin-sensitizing effects	1. Probiotic-like effects	[158]
		2. Induces increased energy metabolism of adipocytes and/or induction of apoptosis, increased expression of neseptin levels in serum		[165,167]
		3. Loss of appetite, reduction in body fat, anti-inflammatory activities, anti-hyperglycemic activity, metabolic and neuroendocrine regulation		[168,169]
		4. Reduces body fat mass by inhibiting adipocyte differentiation through suppression of peroxisome proliferator-activated receptor-γ and by increasing adenosine monophosphate-activated protein kinase resulting in lipolysis		[174]
		5. Enhances the activities of detoxifying enzymes such as glutathione-S-transferase, glutathione peroxidase, glutathione reductase, catalase, and he-oxygenase-1 in the liver and thus suppresses oxidative stress in the liver		[179,180,181]
		6. Blocks the activation of key mediators of cellular inflammation such as NF-κB, 5-lipoxygenase (5-LOX), and cyclooxygenase-2 (COX-2)		[182,183]
		7. Inhibits the activation and proliferation of stellate cells in the liver, which have a known role in the progression of liver fibrosis		[184]
Silymarin		1. Anti-inflammatory, antioxidant, and anti-fibrotic activity		[188]
		2. Reduces insulin resistance		[193]
		3. Reduces liver inflammation by inhibiting lipooxygenase activity and reducing the function of leukotrienes and their effect on Kupffer cells in the liver and by reducing oxidative stress by increasing glutathione levels		
		4. Improvement of serum superoxide dismutase activity and malondaldehyde (MDA) levels in rats in which NAFLD was induced		[192]
		5. Reduce serum aspartate aminotransferase enzyme levels and levels of triglycerides (TGs) and cholesterol including VLDL in NAFLD-induced rats		
Sterculic acid (SA)		1. Improvement in glucose tolerance and blood pressure, reduction in body mass, and benefit in serum levels of triglycerides and adiponectin		[228]
Aquamin [241]		1. Prevents and can even help stop the progression of NAFLD	1. Regulates expression of several proteins related to cell differentiation in the colon mucosa	[235,241]
		2. Reduce the formation of tumors		[236,237]
		3. Reduces inflammation, damage to hepatocytes, and the appearance of collagen deposits	2. Change the intestinal bacterial profile	[241]
Oleic acid		1. Prevents the accumulation of lipids in the liver		[248]
		2. De novo synthesized oleic acid contributes to the protection of hepatocytes against insulin resistance		
		3. Modulates the activity of liver X receptors (LXRs)		[249]
		4. Protects against the cytotoxic activity caused by treatment with palmitic acid-induced steatosis in primary rat hepatocytes in culture.		[259]
Bilirel (BIL)		1. Rapid improvement in liver fat accumulation, improvement in glucose levels and metabolism		[260]
Cannabinoids		1. Decreases fibrosis		[261]
		2. Stimulation of adipocyte metabolism		[262]
		3. Improvement on the insulin–glucose circuit and inhibition of weight gain		[263]
		4. Suppresses the development of NAFLD		[264,265,266]
		5. Decreases hepatic TG synthesis, as does VLDL synthesis, and increases insulin sensitivity		

LDL, Low-density lipoprotein; HDL, High-density lipoprotein; VLDL, Very low-density lipoprotein; TNF-α, Tumor necrosis alpha; NF-κB, Nuclear factor-Kappa beta; ROS, Reactive oxygen species; AST, Aspartate aminotransferase; ALT, Alanine transaminase; NASH, Non-alcoholic steatohepatitis; NAFLD, Non-alcoholic fatty liver disease; TGs, Triglycerides; MDA, Malondialdehyde; IL-1β, Interleukin-1 beta; IL-6, Interleukin-6; 5-LOX, 5-lipoxygenase; COX-2, cyclooxygenase-2.

## 15. Antioxidants and NAFLD

### 15.1. Oxidative Stress and NAFLD

Oxidative stress has a key role in the initiation of NAFLD as well as its development and progression to NASH. As mentioned above, the perturbation of lipid metabolism determines fat accumulation in hepatocytes. In this way, the intracellular organelles such as mitochondria, endoplasmic reticulum (ER), and NADPH oxidase are stimulated to generate acid radicals or reactive oxygen species (ROS). The increased oxidation of the fatty acids and the increased mitochondrial activity stimulate the generation of ROS within the components of the electron transport chain (I, II, and III) in the reaction chain of cytochrome C oxidase. In particular, increased β-oxidation of fatty acids in mitochondria and microsomes appears to generate more ROS in NAFLD [267]. The mitochondria produce ATP through phosphorylation in an oxidative process, and thus superoxide radicals are formed as a byproduct of the oxidative phosphorylation. Similarly, oxidative stress in NAFLD may be caused by the changes that occur in NADPH oxidase and ER stress [268]. Intracellular oxidative stress usually occurs when there is an imbalance between the levels of intracellular ROS and endogenous and enzymatic antioxidants. Clinically, a decrease in endogenous antioxidants has been reported in NAFLD patients [269]. In a clinical study, it was reported that the levels of catalase (CAT), superoxide dismutase (SOD), glutathione peroxidase (GPx), glutathione (GSH), and glutathione reductase (GR) in the serum/plasma of NAFLD patients were disturbed in patients with early and advanced disease characteristics [270]. Increased intracellular ROS provokes changes in insulin sensitivity and the alteration of various essential enzymes involved in lipid metabolism. In liver steatosis, oxidative stress is responsible for triggering immune responses [271]. The experimental and clinical studies demonstrated the infiltration of adaptive immune cells (T cells) into the liver during NASH and the presence of circulating antibodies targeting antigens derived from oxidative stress (Van Herck et al., 2019). In NAFLD, oxidative stress causes the activation of many redox-sensitive transcription factors such as NF-κB and pro-inflammatory mediators (TNF-α), interleukins (IL), etc., leading to liver inflammation, fibrosis, and cell death [272].

NAFLD is a multifactorial disease involving insulin resistance, oxidative stress, and excessive fat intake, and a carbohydrate-based diet that causes the accumulation of excess fat in the liver leading to steatosis [273]. Simple steatosis of the liver can cause intracellular ROS upregulation through induction of the CYP2E1 enzyme. The increased intracellular ROS causes oxidative stress [274]. Accumulations of fat, ROS, and decrease in intracellular antioxidants together cause lipotoxicity, mitochondrial dysfunction, and ER stress in the liver. Fat infiltration in hepatocytes leads to impaired β-oxidation and oxidative phosphorylation in mitochondria, while disturbances in β-oxidation in peroxisomes, and lysosome dysfunction lead to the accumulation of intracellular ROS and hydrogen peroxide radicals [275]. Thus, damaged lipid metabolism is involved in the alteration of oxidative and antioxidant homeostasis, causing redox imbalance and oxidative stress. Impaired lipid metabolism in hepatocytes increases fatty acid uptake via the CD36 transporter, and mitochondrial dysfunction may result in the accumulation of intracellular triglycerides. Redox imbalance in fatty liver increases endoplasmic reticulum (ER) stress by regulating protein response. The chronic stress of the ER and the activation of a prolonged protein response increase the expressions of ER stress proteins such as PKR-like ER kinase, the activation of HNF4α, which is transcription factor 4, a homologous protein that binds CCAAT-enhancer-enhancer, which leads to the activation of pro-inflammatory marker expression and to the activation of cell death pathways in hepatocytes [276]. Furthermore, sustained ER stress leads to activation of the SREBP1c protein to bind sterol elements. Its nuclear translocation can induce the transcription of genes related to lipogenesis. Thus, oxidative stress plays a central role in the initiation and exacerbation of NAFLD.

### 15.2. Antioxidants Effects on NAFLD

Raised lipid peroxidation and dropped antioxidant status have been correlated with NAFLD progression. Thus, oxidative stress is involved in NAFLD progression. For this reason, different antioxidants have been studied experimentally and clinically against NAFLD patients [277]. In the last decade, several clinical and experimental studies have involved oxidative stress in NAFLD conditions and aimed NAFLD with antioxidants. We will further address the effect of some of the entirely studied plant-derived and synthetic antioxidants and antioxidant vitamins to date against experimental and clinical NAFLD conditions. For example, antioxidants such as silybin or silibinin, silymarin vanillin (apocynin), resveratrol, pentoxifylline, and vitamins A, C, and E have come to clinical trials against NAFLD. 

Apocynin, also known as acetonylon, is the natural organic compound structurally related to vanillin (natural vanilla). In a study, CCl4 was orally administrated to rats (1 mL/kg) twice a week for two weeks and they were treated with apocynin (100 mg/kg, orally) daily for two weeks. Apocynin notably lowers serum AST, ALT, and ALP activity and suppresses OS markers (MDA and NO levels) in CCl4-treated rats. Apocynin treatment also recovers catalase and SOD activity in CCl4-treated rats. Thus, apocynin has protective effects in CCl4-induced liver damage by inhibiting lipid oxidation and stimulating the cellular antioxidant system. Synthetic vanillin is a cheap and unhealthy alternative to real vanilla extract. Today, the vast majority of synthetically produced vanillin is made from eugenol or guaiacol, petrochemicals that are often derived from crude oil. Hence, this product is not recommended for use in general and especially not for fatty liver [278].

#### 15.2.1. Bilirel (BIL)

Abenavoli et al. [260] reported the effect of a new antioxidant complex, called Bilirel (BIL) (Pharmaluce, Republic of San Marino), recently introduced on the Italian market. The composition of one pill of BIL was as follows: silymarin 75 mg, chlorogenic acid 3.75 mg, protopine 0.02 mg, L-methionine 75 mg, and L-glutathione 75 mg. They report a case series of seven overweight patients with NAFLD, in which the combination of an Italian Mediterranean diet, increased physical activity, and daily administration of two BIL pills for 6 weeks, resulted in rapid improvement in liver fat accumulation, improvement in glucose levels and metabolism, and weight reduction (Table 2).

#### 15.2.2. Additional Antioxidants

As NAFLD is strongly associated with the presence of oxidative stress, mitochondrial dysfunction, and inflammation, antioxidants can therefore exert a significant ameliorative effect. The products of the plant Silybum marianum, especially silybin, have a specific role in regulating oxidative stress and lipid metabolism. The antioxidant effect of silybin was achieved by activating Nrf-2-related genes, and the lipid-lowering effect was achieved by promoting PPARα, while the anti-inflammatory effect was achieved by inhibiting NF-κB signaling. Resveratrol, a known SIRT 1 and AMPK activator, inhibits SREBP1c, which is responsible for de novo lipogenesis. Resveratrol also inhibits Nrf-2 promoter methylation and protects the NAFLD liver from epigenetic changes.

## 16. Vitamins with Antioxidant Activity in NAFLD

**Figure 2:**
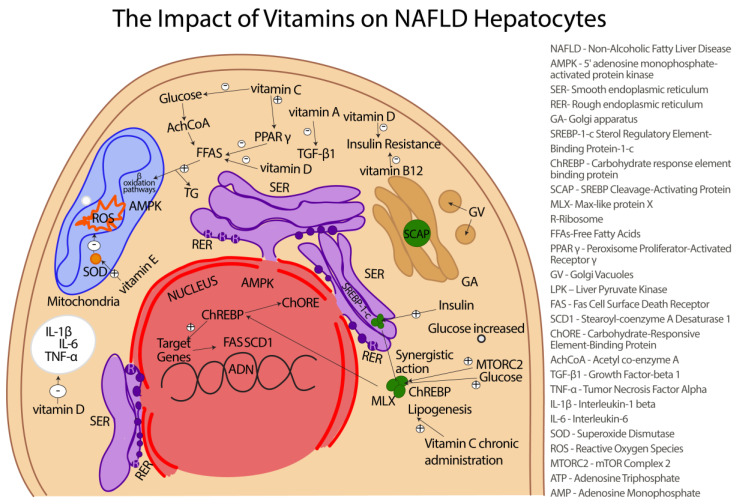
The impact of vitamins on hepatocytes affected by NAFLD.

Vitamins control various fundamental enzymatic processes in the liver, and modifications in the metabolism of vitamins have a crucial role in the progression of NAFLD. Vitamins A, C, and E have been particularly studied in relation to NAFLD due to their antioxidant activity. Likewise, serum concentrations of vitamins D and B12 have been reported to have a strong correlation with NAFLD severity [279] (Figure 2). Hepatocyte cells (HSC) store most of the body’s retinol [280]. 

### 16.1. Vitamin A and NAFLD

At the same time, the impaired metabolism of Vitamin A caused its accumulation in hepatocytes and not in HSC in mice in which NAFLD was induced. Thus, NAFLD causes the amassment of Vitamin A in hepatocytes, which may provoke disease progression [281]. Retinoic acid (RA) treatments have been demonstrated to be effective and antioxidant by lowering mitochondrial ROS and enhancing SOD2 in mice (Table 3). Retinoic acid treatment also increased the expression of hepatic Sirt1 and inhibited SREBP1c expression in HFD-fed mice [282]. Moreover, through signaling regulation of lipid metabolism involving Retinoid X receptors alpha (RXRsα), the liver could be protected by RA. In this sense, the increased concentrations of circulating RA protect against hepatic steatosis itself, as well as against liver damage in NAFLD populations [283]. A considerable presence of lipid droplets in stellate cells of the liver can affect the release of retinol. It is known that once released, it converts into retinoic acid with a beneficial role regarding inflammation, fibrogenesis, and carcinogenesis. This process is closely related to the triggering of a pro-inflammatory and pro-fibrogenic phenotype in hepatic stellate cells [284] (Table 3). A reduction in the release of transforming growth factor-beta 1 (TGF-β1) induces the suppression of hepatic stellate cells, and ultimately of fibrogenesis caused by NAFLD due to the ability of RA to inhibit proto-oncogene tyrosine-protein kinase MER (MERTK) in Kupffer cells. Thus, the modulation of RA release may represent a common genetic pathway associated with NAFLD [285] (Figure 2).

### 16.2. Vitamin C and NAFLD 

In vivo Vitamin C supplementation reduces the hepatic fatty acid load by promoting the gene expression of PPARα-dependent β-fatty acid genes in in mice fed HFDs and in which NAFLD is induced [286] (Table 3). It can be emphasized that the prophylactic treatment of Vitamin C (15 and 30 mg/kg/day) significantly dropped body weight and steatosis, and thus induced a decrease in the risk of NAFLD in mice. In a therapeutic study, the administration of 30 mg/kg/day of Vitamin C reduced steatosis and NAFLD in mice. However, the administration of Vitamin C overdoses (90 mg/kg/day) did not reduce the risk of NAFLD development. In fact, high-dose Vitamin C intake significantly increased body weight, inflammation, and adipose tissue mass. [287]. This study clearly shows that an accurate dose of Vitamin C must be determined in cases of NAFLD. In NAFLD rats with choline deficiency, the administration of Vitamin C (30 mg/kg/day) significantly suppressed steatosis and oxidative stress. In the MCD diet-induced NASH model, a megadose administration (2.5 g/kg/day) of Vitamin C reduced macro-vesicular steatosis. However, AST and ALT were also increased after overdose Vitamin C administration [122].

In contrast, a recent study reported that Vitamin C deficiency leads to the inhibition of NAFLD. Vitamin C-deficient knockout mice reduced NAFLD progression compared to control mice. Vitamin C-deficient mice exhibit increased levels of the transcription factor -SREBP-1c and decreased expression of FAS [288], suggesting that long-term Vitamin C deficiency may be useful for inhibiting de novo lipogenesis via the SREBP-1c protein. However, NAFLD inhibition mediated by Vitamin C deficiency should be carefully investigated. There are reports according to which long-term Vitamin C is beneficial by improving adiponectin and reducing liver TG level, and therefore the chances of NASH in NAFLD patients (Figure 2).

Wei et al., 2016, demonstrated that, in the middle-aged male population without obesity, there is a significant inverse association between ingested Vitamin C and NAFLD. [289]. They explained that estrogens are responsible for these sex differences because their beneficial roles against NAFLD in middle-aged women may inhibit Vitamin C effects [290]. A strong causal relation has been demonstrated between obesity and NAFLD. Regarding the relationship between Vitamin C, obesity, and NAFLD (Table 3), Ipsen et al. have speculated that the therapeutic effects of this vitamin can be counteracted by obesity [291]. 

### 16.3. Vitamin E and NAFLD 

Regarding Vitamin E, it was demonstrated that three weeks of Vitamin E administration (0.5 g/kg) to HFD-induced phosphatidylethanolamine N methyltransferase-deficient NAFLD mice induced the normalization of cholesterol metabolism as well as reduced the inflammation and fibrosis associated with oxidative stress; however, it failed to reduce liver TG content [292] (Table 3). Additionally, Vitamin E administration reduced fructose diet-induced NAFLD by stimulating the Nrf2/carboxylesterase 1 pathway implicated in lipogenesis [293]. Therefore, as a strong antioxidant, Vitamin E has been extensively studied as an adjuvant along with other medicines against NAFLD pathology, but there are no unequivocal conclusions for human use. In mice who received a diet poor in methionine-choline (MCD), Vitamin E, due to decreasing liver markers and decreasing histological steatosis, can be considered an inhibitor of steatohepatitis [294]. As a result, SOD activity increased and malonaldehyde (MDA) concentration decreased. In addition, the genes responsible for fibrosis, inflammation, and apoptosis were inhibited. Additionally, the activation of hepatic stellate cells and the replenishment of hepatic glutathione are also therapeutic effects of Vitamin E in mice with NAFLD [295]. Abdel-Maboud et al. [296] found that, depending on the dose of Vitamin E, the levels of some liver markers such as ALT and AST improve. Among these, the most significant variation was observed in AST activity, with adequate repercussions on the NAFLD activity score (NAS). Moreover, this process strongly influenced anthropometric parameters such as weight and IMC [296]. As previously mentioned, the increase in oxidative stress is one of the most common changes in NAFLD. This result is represented by the accumulation of reactive oxygen species, concomitantly with the incapacity of the body to defend itself through the antioxidant systems, which ultimately leads to damage to DNA and tissues [297]. In this sense, it is known that Vitamin E is one of the most powerful antioxidants [298]. These effects are observed at the molecular level through cellular, biochemical, genetic, and signaling pathways, with results in the modulation of the inflammatory response and cell proliferation. Moreover, it is responsible for regulating the cellular signaling of different enzymes essential in molecular signal translation, such as 5-lipoxygenase, cyclooxygenase-2 (COX-2), protein kinase C (PKC), and protein phosphate 2A (PP2A) (Table 3). Additionally, there are certain factors such as mitogen-activated protein kinase (MAPK) that can be modulated by Vitamin E [299]. Wang et al. demonstrated that Vitamin E reduces liver fibrosis by suppressing TGF-β expression, [294]. In addition, due to its capacity to stimulate the activation and transcription of PPARγ in adipocytes in mice, Vitamin E stimulates the expression of adiponectin (Figure 2). It is well documented that the protein–hormone adiponectin is involved in the regulation of glucose concentration and the degradation of fatty acids [300]. The effects of Vitamin E on gut microbiota in NAFLD are discussed in several studies and seem to change the gut microbiota composition into a healthier one [301]. Similarly, in mice models in which colitis was induced, the changes provoked by Vitamin E administration were observed through favorable modifications in intestinally disturbed microbiota by the increases of portal LPS [302] (Table 3). 

### 16.4. Vitamin D and NAFLD

Like all organs, the liver has its own typical immunity configuration. The components that belong to innate immunity and influence the pathology of NAFLD are represented by Kupffer cells, hepatic stellate cells, and natural killers. Besides these cells, there are macrophages and monocytes involved [303]. Recruited macrophages and Kupffer cells have been associated with insulin resistance and NASH, since they synthesize the well-known pro-inflammatory cytokines interleukin-1 beta (IL-1β), IL-6, and TNF-α, [304]. Regarding macrophages, they are divided into M1 and M2, the difference consisting of the fact that the M1 macrophages are classically activated, whereas the M2 macrophages are alternatively activated [305]. The polarization and dysregulation of M1-like/M2-like macrophages, in which M1-like initiate and sustain inflammation, whereas M2-like attenuate chronic inflammation, are linked to NAFLD pathology. Finally, all these processes determine insulin resistance and some metabolic diseases such as diabetes and obesity [304]. Due to the fact that liver cells express receptors for Vitamin D (VDR), the liver can be protected against the inflammation induced by chronic hepatitis following the administration of Vitamin D [306] (Figure 2). Moreover, by stimulating VDR expression, insulin sensitivity increases. This process involves the Glut-4 translocators of muscle cells which therefore transport glucose inside the cell and then decrease blood glucose concentration. In addition, the modulation of free fatty acids (FFAs) is associated with the improved expression of these receptors [307]. Additionally, Vitamin D treatment decreases the cytokeratin 18-associatedapoptotic fragment M30 and thus reduces liver damage; therefore, Vitamin D exerts anti-fibrotic, anti-inflammatory, and anti-cirrhotic effects [308,309]. Sharifi et al. demonstrated that tumor necrosis factor alpha (TNFα) and C-reactive protein were significantly affected by Vitamin D administration [310]. In the pathogenesis of NAFLD, insulin resistance is very critical and is considered a significant risk factor associated with NAFLD. As previously described, it is linked to the increase in oxidative stress and lipotoxicity [311]. Nuclear factor κ-β (NF-κB) represents a mediator through which, if activated, a pro-inflammatory modulation can be made, resulting in the release of pro-inflammatory cytokines such as IL-1β, IL-6, or TNF-α. In this way, Kupffer cells are finally activated [312]. Additionally, histological features of NASH can be induced by the activities of these cytokines. In humans, the increased gene expression of these cytokines is much more frequent in the liver of patients with NASH compared to the normal liver of obese patients. The higher the secretion of these cytokines, the greater the severity of NAFLD [313]. In this regard, Neyestani et al. 2012 observed that, in type 2 diabetic patients, supplementing a daily intake of 1000 IU of Vitamin D single or combined with calcium, for 12 weeks, led to a decrease in IL-1β and IL -6 pro-inflammatory cytokines secretion [314] (Table 3). In other studies, after 10 weeks of administration of 50,000 IU Vitamin D weekly, a notable decrease in TG was shown in patients with NAFLD [315] (Figure 2). Interestingly, regarding transaminases, Amiri et al., [316] discovered that the combination of 25 μg/day Vitamin D with 500 mg calcium carbonate during 12 weeks, decreases ALT and AST activities compared to patients who were administered only Vitamin D or the placebo group. Different studies have shown that the dietary intake of calcium influences the absorption of Vitamin D and through this changes the blood lipid profile. Thus, after 12 weeks of simultaneous administration of Vitamin D together with calcium, the ratios of LDL-C/HDL-C, and TC/HDL-C decreased. Concomitantly, non-HDL-C increased [317]. 

At the liver cells level, depending on the time and the administered dose, vitamins can have a positive or negative impact. Vitamin A can inhibit the release of the cytokine TGF-β1. Chronic administration of low doses of Vitamin C exerts antioxidant effects by suppressing ROS release. It stimulates PPAR γ, which in this way decreases the concentration of FFAs. The same effect is observed in the case of Vitamin D. However, chronic administration of high doses of Vitamin C activates lipogenesis, which ultimately leads to NAFLD. Another beneficial vitamin in this disease is D, which increases insulin sensitivity. Additionally, Vitamin B12 has the same properties. Vitamin E, known for its role in the fight against ROS, has stimulating effects on the activity of the SOD enzyme.

### 16.5. Vitamin B12 and NAFLD

DNA synthesis and repair are dependent on appropriate levels of Vitamin B12 [303]. Likewise, the disruption of mitochondrial metabolism, involved in the pathology of NAFLD, is influenced by Vitamin B12 [318]. Only a few studies evaluated the effect of Vitamin B12 and NAFLD. Even so, the results were contradictory. There are studies that discovered an association between NAFLD and hyperhomocysteinemia [319]. This can be demonstrated by the fact that intracellular lipid metabolism is altered at high serum concentrations of homocysteine, which in the hepatocyte causes in the endoplasmic reticulum an increase in oxidative stress. This affects the signaling pathway in response to sterols, and as a consequence, the progression of NAFLD occurs [319]. In this way, the daily administration of 1000 µg of cyanocobalamin for 3 months in NAFLD patients decreased the serum concentration of homocysteine [320]. In NAFLD patients, by administering Vitamin B12, the serum concentration of homocysteine decreased significantly. Therefore, Vitamin B12 supplementation exerts therapeutic effects regarding the pathology of NAFLD [320]. Moreover, in NAFLD pathology, it has been observed that MDA, which is a marker of lipid peroxidation, is always increased [321]. After the administration of Vitamin B12, the serum concentration of MDA decreased significantly. It is believed that the basis of this change could be the fact that Vitamin B12 lowers the levels of homocysteine, and in this way, changes the metabolism of MDA, in the sense of the decrease. There are studies that confirm this speculation since it was demonstrated that supplementation with folate or B12 decreased induced decrease in MDA activity [322] (Table 3). Unfortunately, it is not known whether Vitamin B12 action is direct or indirect. Regarding insulin resistance, it was observed that treatment with Vitamin B12 reduces fasting blood glucose (FBG) (Table 3). An inversely proportional relationship was observed in the study of Al-Daghri et al. [323] between Vitamin B12 and FBG [323]. Moreover, other studies have reported [324] that the combined daily administration of B12 (500 µg) with folic acid (5 mg) significantly improves the Homeostatic Model Assessment for Insulin Resistance (HOMA-IR), as well as FBG. The results are not very relevant because the vitamins therapy was not performed separately. Additionally, in this case, the mechanisms underlying the improvement of the carbohydrate profile target homocysteine [325].

**Table 3:** Impact of micronutrients on NAFLD.

Type of Micronutrient	Effects on Liver	Effects on Intestinal Microbiota	References
Se	1. Administration of a combination of Se and zinc improved the lipid profile, liver functions, and liver steatosis in rats		[206]
	2. Se and probiotics reversed the negative effect of feeding in mice on HFD and improved liver functions and steatosis		[207]
	3. Decreases the number of hepatic stellate cells (HSCs) and liver fibrosis induced by CCl4 treatment in mice		[209]
Vitamin A	1. Retinoic acid (RA) administration has been shown to be an effective antioxidant by reducing mitochondrial ROS and by increasing SOD2 in mice		[282]
	2. Protects the liver against hepatic steatosis itself, as well as against liver damage in NAFLD populations		[283]
	3. Reduces the release of transforming growth factor beta 1 (TGF-β1)		[285]
	4. Suppresses the activation of hepatic stellate cells and fibrogenesis		
Vitamin C	1. Reduces hepatic fatty acid load by promoting the gene expression of PPARα-dependent β-fatty acid genes in HFD-induced NAFLD mice		[286]
	2. Attenuates steatosis and NAFLD in mice		
	3. Improves adiponectin levels and reduces liver TG levels and thus prevents NASH progression in NAFLD patients		[288]
	4. Significant inverse association between ingested Vitamin C and NAFLD		[289]
Vitamin E	1. Normalizes cholesterol metabolism and reduces inflammation and fibrosis associated with oxidative stress		[292]
	2. Attenuates fructose diet-induced NAFLD by activating the Nrf2/carboxylesterase 1 pathway involved in lipogenesis		[293]
	3. Is considered an effective inhibitor of steatohepatitis		[295]
	4. Inhibits the expression of genes responsible for fibrosis, inflammation, and apoptosis		[296]
	5. Improves the blood levels of ALT and AST		[299]
	6. Responsible for regulating the cellular signaling of different enzymes essential in molecular signal translation, such as 5-lipoxygenase, cyclooxygenase-2 (COX-2), protein kinase C (PKC), and protein phosphate 2A (PP2A)		[300]
	7. Stimulates the expression of adiponectin		[302]
	8. Induces favorable modifications in intestinal disturbed microbiota by the increases of portal LPS		
Vitamin D	1. Protects the liver against the inflammation induced by different chronic hepatitises		[306]
	2. Increases insulin sensitivity		[307]
	3. Induces anti-fibrotic, anti-inflammatory, and anti-cirrhotic properties		[308]
	4. Decreases secretion of the pro-inflammatory cytokines IL-1β and IL-6		[314]
	5. Decreases TG levels in NAFLD patients		[315]
	6. Improves blood lipid profile		[317]
Vitamin B12	1. Affects the disruption of mitochondrial metabolism, involved in the pathology of NAFLD		[318]
	2. Therapeutic effects regarding the pathology of NAFLD		[320]
	3. Significantly decreases the serum concentration of MDA		[322]
	4. Reduces fasting blood glucose (FBG)		[323]

LDL, Low-density lipoprotein; HDL, High-density lipoprotein; VLDL, Very low-density lipoprotein; TNF-α, Tumor necrosis alpha; NF-κB, Nuclear Factor-Kappa beta; ROS, Reactive oxygen species; AST, Aspartate aminotransferase; ALT, Alanine transaminase; NASH, Non-alcoholic steatohepatitis; NAFLD, Non-alcoholic fatty liver disease; TGs, Triglycerides; MDA, Malondialdehyde; IL-1β, Interleukin-1 beta; IL-6, Interleukin-6; 5-LOX, 5-lipoxygenase; COX-2, cyclooxygenase-2; PKC, Protein kinase C; PP2A, Protein Phosphate 2A; LPS, lipopolysaccharide; PPARα, Peroxisome Proliferator Activated Receptor alpha.

In conclusion, when we take vitamins, we must take into account several aspects, namely the dose, the physiological and pathological state of humans, and the duration of the treatment. 

## 17. Bile Acids and NAFLD

Regarding fat absorption, bile acids are essential [326]. They are obtained through the oxidation of cholesterol in hepatocytes. The process involves several stages and results in primary bile acids such as chenodeoxycholic and cholic acids. The synthesis pathways of primary cholic acids are defined as classical (75%) or alternative (25%) [327]. The primary bile acids obtained are then conjugated with taurine or glycine. These acids are water-soluble when entering the duodenum at a pH of 3–5. Then, they are capable of emulsifying and solubilizing fats [328]. The bile ducts store bile acids via the bile salt export pump. Then, through a biliary tree system, the bile with newly formed bile acids is stored in the gallbladder during the inter-digestive periods. During the digestive period, through the contraction of the gallbladder, the bile is transported into the duodenum [329]. Primary bile acids are converted into secondary acids (lithocholic acid, deoxycholic acid, and ursodeoxycholic acid) by the intestinal microbiota [330]. Enterocytes from the distal ileum carry out the reabsorption of bile acids in a proportion of over 95%. Then, they are transported back to the liver. The intestinal microbiota is responsible for the deconjugation of the remaining 5%, which is then excreted in the feces. However, a very small amount reaches the peripheral tissues, where it causes peripheral effects [329]. 

Additionally, bile acids are considered signaling molecules in charge of regulating glucose and lipid metabolism. This is achieved by activating several factors such as Takeda G protein-coupled receptor 5 (TGR5), farnesoid X receptor (FXR), and multipurpose nuclear receptor. In NAFLD patients, a disturbance of bile acid signaling as well as dysbiosis is observed [331]. The stimulation of bile acid signaling pathways regulates glucose and lipid homeostasis by changing the intestinal microbiota, resulting in the improvement of the metabolic phenotype [332]. The intestinal microbiota are characterized by four types of bacteria: *Actinobacteria*, *Bacteroidetes*, *Firmicutes,* and *Proteobacteria* [333]. The imbalance determined by the intestinal microbiota is called dysbiosis. Obesity is associated with an increase in *Firmicutes* and a decrease in *Bacteroidetes*, which results in the stimulation of dietary energy gain [334]. It was demonstrated that colonizing mice with a specific microbiota from obese individuals had as a result an increase in body fat. In addition, mice fed a Western diet had a lower *Bacteroidetes*/*Firmicutes* ratio in the distal intestine [335]. The influence of bile acids on the intestinal microbiota can be direct or indirect, when it is mediated by FXR. The acid with the greatest antibacterial influence on the intestinal microflora is deoxycholic acid [330]. Moreover, the antibacterial action of non-conjugated acids is much stronger compared to that of conjugated acids [336]. In addition, the activation of FXR results in the stimulation of peroxisome proliferator-activated receptor alpha (PPAR-alpha). This nuclear receptor regulates the metabolism of glucose, lipids, and anti-inflammatory activity [337]. Thus, bile acids via FXR activation can reduce TG concentration through a signaling mechanism involving sterol regulatory element-binding protein 1 (SREBP-1) and its small heterodimer partner (SHP). This modulates the transcription of lipogenic genes [338]. Thus, from what can be seen, the most important mechanism for the development of NAFLD is the damage to the acid bile-signaling pathways [325].

## 18. Imbalance of the Intestinal Microbiota, Choline Metabolism, and NAFLD 

In the causal link that exists between NAFLD and the imbalance of the intestinal microbiota, the increase in the permeability of enterocytes, the energy from food, the decrease in the metabolism of choline, the excessive growth of intestinal bacteria, and finally, the suppression of the metabolism of bile acids can be highlighted. The most well-known mechanism involved in the deregulation of the intestinal microbiota with repercussions on NAFLD includes the amplification under the action of this destabilization of the energy from the diet. It has been shown that the synthesis of short-chain fatty acids is much higher in obese patients. These acids are the main fermentation product of the bacteria that metabolize dietary fibers [334]. Thus, the total energy produced by the diet is increased, due to the bacterial metabolism of fibers, which in physiological conditions would be eliminated through defecation [339]. Additionally, increased intestinal permeability is closely associated with NAFLD. Enterocytes are essential for adequate intestinal immunity. Between some pathogens and the lamina propria, intestinal cells represent a physical barrier. These cells communicate with each other through junctions. The dysregulation of the intestinal microbiota can be closely linked to certain signaling pathways, which can regulate the expression and distribution of proteins and thereby intestinal permeability [340]. Regarding the increased permeability, this is responsible for triggering an inflammatory response in the liver through the receptor for bacterial DNA (TLR9) and receptor for bacterial flagellin (TLR5), due to the fact that translocation of bacterial components takes place in intestinal cells [341]. The increase in enterocyte permeability, as well as the multiplication of bacteria, in NAFLD patients, was correlated with the severity of steatosis [342]. It is known that LPS is closely related to the TLR4/NF-κB signaling pathway [343]. 

The role of choline in the pathogenesis of NAFLD is major. It has been observed that the deficiency of choline and methionine is correlated with the increase in the release of interleukins IL-1β, hepatic inflammation, and finally with the occurrence of NAFLD [344]. This is possible due to the fact that phosphatidylcholine results from choline metabolism, which is involved in the degradation of lipids in the liver [345]. Moreover, there is an inversely proportional relationship between supplementing food intake with choline and NAFLD [346].

## 19. The Activity of the Enzyme AMPK (5′ Adenosine Monophosphate-Activated Protein Kinase) in NAFLD

Considering that most therapies for NAFLD have focused on caloric restriction and sports [347], treatment strategies have been proposed to improve this disease. The majority of the strategies are based on AMP-activated protein kinase (AMPK), which is linked to different metabolic mechanisms. Furthermore, many of the effects of therapeutic compounds are mediated by the modulation of AMPK activity [348,349].

AMPK is a heterotrimer consisting of two regulatory units (β and γ) and one with catalytic activity [350]. Each of the three subunits have several isomers. AMPK is an enzyme considered crucial in the maintenance of energy balance. It is activated by different stimuli that mainly lead to the consumption of ATP. Between both, there is a relation of inverse proportionality. This explains the activation of AMPK under physical and cellular stress conditions, in which ATP production is considerably low or ATP consumption is increased (physical activity) [351]. AMPK, in the liver, is controlled by liver kinase B1 (LKB1). In NAFLD patients, even if the ATP content is reduced, the activity of AMPK is altered [352]. 

In NAFLD, increasing the activity of AMPK can inhibit the synthesis of fatty acids and cholesterol by downregulating the expression of the adipogenesis gene. Simultaneously, by increasing the expression of fatty acid oxidation and lipid decomposition genes involved in fatty acid oxidation and lipid decomposition, the body’s natural lipid balance can be maintained. 

Secondary factors also play an essential role in the control of AMPK activity. The most plausible explanation for this can be related to the fact that LPS and TNFα (inflammatory factors) can decrease the activity of AMPK [353]. 

At present, some AMPK activators are thought to be beneficial during adequate treatment. Therefore, the activation of the AMPK signaling pathway is a potential therapeutic target for disorders of the liver. In the pathology of NAFLD, the increased concentration of FFAs is the most important factor contributing to the increase in hepatic lipids [354]. Therefore, the control of the factors involved in the regulation of their metabolism represents an aspect that must be taken into account. Insulin is one of them, because, in healthy people (sensitive to insulin), it suppresses lipolysis in white adipose tissue. In this respect, in NAFLD patients who also have insulin resistance, the insulin loses its suppression capacity. The result is the release of FFAs into circulation. Considering that FFAs reach the portal circulation directly, the effect is more dangerous because they make deposits in the form of visceral fat [355]. In addition, a part of FFAs reach the liver, and under esterification they form TGs, leading to an increase at the liver level. However, the hepatic esterification process is independent of insulin and dependent on the concentration of FFAs [356]. Insulin resistance is correlated with reduced AMPK activity in obese persons. In this sense, AMPK in adipose tissue is a very important factor for NAFLD [357]. Mottillo et al., 358 in a knockout mouse model showed that the ablation of AMPK activity in adipose tissue is correlated with increased insulin resistance and lipid accumulation in the liver [358]. 

Moreover, AMPK from adipose tissue macrophages can inhibit different signaling pathways such as TNF-α and IL-1β (Table 4). This is why AMPK activation has been proposed to suppress inflammation [359] (Figure 2). Due to the fact that AMPK is responsible for suppressing primary inflammatory lesions and HSCs, it is used to mitigate fibrosis [360]. Thus, by phosphorylating transcription factors such as ChREBP and SREBP-1c, AMPK inhibits the transcription of lipogenic genes [361] (Table 4).

Contrary to what is believed, an excess in amino acids (AAs, high-protein diet) has been shown to inhibit AMPK activity [362]. Increased protein intake reduces AMPK phosphorylation. In the liver and hypothalamus, mTOR phosphorylation is increased. Thus, the increased concentration of AAs decreases the AMP/ATP ratio. In this way, AMPK activity is suppressed [363]. Moreover, AMPK improves NAFLD by stimulating metabolism at the mitochondrial level and intensifying the oxidation of fatty acids in the liver [364] (Figure 2).

**Table 4:** The effects of different enzymes on NAFLD.

Type of Enzyme	Positive Effects of Enzymes and/or Supplements on Liver	Adverse Effects of Enzymes Inhibitor Treatments	References
Stearoyl-CoA desaturase 1 (SCD1) inhibitors	1. SCD1 inhibitors are associated with amelioration of NAFLD, diabetes, dyslipidemic failure, and hepatitis C virus infections	1. Knockout mice develop multiple skin eczemas accompanied by weight disturbances [223]	[220,223]
	2. SCD1 inhibitors reduces the accumulation of triglycerides in the liver of rats suffering from NASH by 80%	2. Failure of thermoregulation, transepidermal water loss, and metabolic problems [224]	[222]
	3. Attenuate the increase in aspartate aminotransferase (AST) enzyme levels and alanine transaminase (ALT) by 86% and 78%, respectively	3. Cause atrophy of sebocyte cells (epithelial cells in the skin), effects reflected in hair loss and dry eyes causation	[226]
AMPK (5′ adenosine monophosphate-activated protein kinase)	1. Inhibits different signaling pathways such as TNF-α and IL-1β		[359]
	2. Suppresses inflammation		[361]
	3. Inhibits the transcription of lipogenic genes		[364]
	4. Improves NAFLD by stimulating metabolism at the mitochondrial level and intensifying the oxidation of fatty acids in the liver		

NASH, Non-alcoholic steatohepatitis; NAFLD, Non-alcoholic fatty liver disease; TNF, Tumor necrosis factor alpha; IL-1β, Interleukin-1 beta.

## 20. Cannabinoids and NAFLD

Since many nutritional and pharmacological therapies have been used to treat NAFLD, there are additional compounds that must be considered. One of them is the endocannabinoid system (ECS), which represents a physiological complex. The endocannabinoid system (ECS) represents an endogenous signaling system demonstrated to play a key role in the regulation of appetite, metabolic processes, and energy balance. The ECS is made up of bioactive lipids, the endocannabinoids, enzymes that regulate their production and degradation, and receptors through which they transmit their signal [365]. The most studied and well-known receptors of the ECS are the G-protein-coupled receptors: cannabinoid receptor 1 (CBr1) and 2 (CBr2). CBr1 is mainly expressed, but not limited to, the central nervous system (CNS) and centrally and peripherally regulates metabolic homeostasis. CBr2, on the other hand, is mainly expressed by immune cells and plays a role in inflammation processes [366]. It is well established that obesity is associated with dysregulation of the ECS, resulting in high endocannabinoid “tone”, which in turn leads to increased appetite, lipogenesis, adipogenesis, and decrease in energy expenditure, which further exacerbates adiposity, thus creating a vicious cycle. In addition to CB1 and CB2, endocannabinoids can also be related by other receptors such as G protein-coupled receptor 55 (GRP55) and peroxisome proliferator-activated nuclear receptors (PPARs). Therefore, the independent effects promoted by cannabinoids may be related to their high lipophilicity. 

Moreover, in the endocannabinoid system there are other important compounds such as exocannabinoids or phytocannabinoids. They are used as a treatment for different metabolic diseases [367]. The most studied compounds from *Cannabis* sp. are delta-9-tetrahydrocannabinol (THC), cannabidiol (CBD), followed by tetrahydrocannabivarin (THCV). Even if they were performed on in vivo models in terms of the effects of their studies in certain metabolic pathologies [368], only a few studies focused on their effects on NAFLD patients [369]. 

Regarding the endocannabinoid system, there are studies in which CB1 and CB2 in animals, as well as in cell cultures, had fibrosis-stimulating effects. At the same time, CB2 had a pro-inflammatory effect and determined insulin resistance [370]. Insulin resistance is linked to the modification of CB2Rs-dependent signaling pathways, through a different mechanism than CB1Rs [264]. 

Animal studies have shown that decreasing food intake and increasing energy consumption are correlated with the antagonism of CB1. This effect results in the improvement of different metabolic diseases [365]. In addition, the stimulation of adipocyte metabolism is correlated with the reduction in CB1 activity in visceral fat [371]. 

In human cell cultures, the CB1 antagonist decreases fibrosis [261]. It is important to note that, regarding phytocannabinoids, their effects on different organs can be different because they can interact with each other. An important example relates to the fact that CBD can antagonistically affect THC through different CBR1 and non-CBR1 receptor-related mechanisms of action. Other effects can be complementary or synergistic [372]. Regarding the medicinal effects of this plant, the most important is the ratio THC: CBD: THCV [373]. In addition to the previously mentioned compounds, there are also precursor compounds such as cannabidiol acid (CBDA), ∆9 tetrahydrocannabinol acid (THCA), and tetrahydrocannabivarin acid (THCVA). They are recognized for their therapeutic properties in obese mice, such as their improvement effects on the insulin–glucose circuit and inhibition of weight gain [262] (Table 2). The dysregulation of the endocannabinoid system is one of the main factors in the development of NAFLD. It is expected that the consumption of cannabis will increase in the next years due to its therapeutic properties in different pathologies. However, it is known that the majority of metabolic disorders that lead to the development of NAFLD are associated with chronic consumption of cannabis. Additionally, it is well established that chronic consumption of cannabis causes an increase in appetite as well as in ingested calories [374]. In addition, with the increasing appetite, a large number of unhealthy foods rich in fats and refined carbohydrates are consumed. We cannot neglect the fact that there are many studies which have shown the therapeutic effects of cannabis suppressing the development of NAFLD (Table 2). In this sense, chronic consumption was associated with a decrease in the prevalence of metabolic syndrome and type 2 diabetes [263]. Moreover, chronic marijuana use has been associated with lower obesity among users [375]. The antagonistic action of CBD and THCV on CB1R may be one of the mechanisms by which cannabis consumption decreases the prevalence of NAFLD as well as other metabolic pathologies [376]. Through CB1R antagonism, hepatic TG synthesis decreases [264], as does VLDL synthesis [266] while insulin sensitivity increases [265] (Table 2). By improving IR, hepatic glucose homeostasis is restored, fat deposits decrease, and therefore cannabis inhibits the development of NAFLD [377]. Additionally, phytocannabinoids have anti-inflammatory roles. They may decrease adipokines and different cytokines, such as IL-6 and TNF-a. This can lead to NF-kB upregulation [378]. Another beneficial mechanism for NAFLD regarding cannabis consumption is represented by the ability of THC to downregulate CBR1 and develop tolerance to these receptors. In this way, an inversely proportional relationship between marijuana consumption and NAFLD develops [379]. In conclusion, cannabinoids may suppress the development of NAFLD.

## 21. Conclusions

We can conclude that, even though the mechanisms associated with NAFLD initiation and progression remain not fully deciphered in this review, we address a number of factors demonstrated to be involved in NAFLD including genetic and epigenetic modifications, signaling, molecular, and biochemical factors, effects of dietary factors, effects of microbiota, and additionally we address the effects of behavioral factors. All these effects can be related from the “two hits” hypothesis to the current “multiple parallel hits hypothesis”. In this context, we present specific compounds and the mechanisms associated with their effects which can ultimately prevent or diminish the progression of NAFLD. In this regard, we concentrate on the following: 

1. Potential bioactive nutrients that may interfere with NAFLD. In this sense, dark chocolate, cocoa butter, and peanut butter may be involved in decreasing cholesterol concentration. Moreover, dark chocolate can affect glucose metabolism by reducing oxidative stress. Additionally, cocoa butter has anti-inflammatory effects. Usually, this pathology is accompanied by insulin resistance, which over time perpetuates with reaching type 2 diabetes. That is why it is important to know how it can be prevented and/or efficiently fought against. 

2. In addition to physical exercise, the sweeteners used in coffee and other frequent beverages also play an important role. On the one hand, most of them are consumed without knowing their effects on the body. For this reason, is necessary to know that some sweeteners have negative effects on the intestinal microbiota, others have no effects, and some have beneficial effects. Thus, stevia has proven to be adequate for improving carbohydrate metabolism, liver steatosis, and liver fibrosis. Moreover, it can decrease inflammation through the suppression of oxidative stress. Maltitol is used to prevent obesity, hyperglycemia, hypercholesterolemia, and fatty liver degeneration. Another sweetener widely used is Erythritol, which may have a role in alleviating NAFLD.

3. On the other hand, there are adequate compounds demonstrated to exert beneficial actions on NAFLD. Most of them are used for therapeutic purposes in NAFLD. In order to lower the serum concentration of triglycerides, glutathione, soy lecithin, silymarin, Aquamin, and cannabinoids are used. In addition, turmeric and curcumin extracts have antioxidant, anti-inflammatory, and anti-fibrotic properties, as well as insulin-sensitizing effects. Bilirel decreases liver fat accumulation and improves glucose metabolism. Additionally, serum superoxide dismutase activity and malonaldehyde (MDA) levels are improved by silymarin.

4. We additionally address the effects of micronutrients, especially vitamins. The effects of micronutrients directly depend on the dose administered. Even if most studies demonstrate the beneficial role of vitamins in the pathology of NAFLD, there are exceptions. Thus, the widely used Vitamin C, especially in overdose during the COVID-19 and post-COVID-19 period, is no longer beneficial. In fact, high-dose Vitamin C administration significantly increased body weight, adipose tissue mass, and inflammation. Regarding the administration of selenium, even if in most studies there is a positive correlation between it and NAFLD, there are reports that indicate negative effects. A Se-enriched diet provoked hepatic nodular hyperplasia in rats. Vitamin A has a protective role against mitochondrial ROS and may suppress fibrogenesis. Vitamin E seems to alter the gut microbiota composition into a healthier one. Vitamin D exerts anti-fibrotic, anti-inflammatory, and anti-cirrhotic effects. Vitamin B12 significantly decreases the serum concentration of MDA.

5. Additionally, the activity of some enzymes is related to the pathology of NAFLD. For example, SCD1 inhibitors are recognized for their beneficial effects on NAFLD, diabetes, dyslipidemic failure, and hepatitis C virus infections. Specifically, they can significantly decrease the accumulation of triglycerides in the liver of rats suffering from NASH. As one of the key enzymes in human metabolism, AMPK can suppress inflammation by inhibiting different signaling pathways such as TNF-α and IL-1β. Moreover, it inhibits the transcription of lipogenic genes. 

Having mentioned these, we conclude that NAFLD can be prevented or improved by different factors through their involvement in the signaling, genetic, and biochemical pathways that underlie NAFLD. Therefore, exposing this vast knowledge to the public is particularly important. 

As future perspectives, through the information that this work provides, we hope that it can be used by clinicians as a therapeutic nutritional alternative, starting from the premise that an adequate diet can prevent, in healthy individuals, the occurrence of NAFLD. In the case of patients with this disease, proper nutrition can delay, and even suppress, its progression and development, as well as collateral diseases such as type 2 diabetes or cardiovascular diseases.

In addition, we believe that this paper contains information that could represent a sustainable alternative to public health policy. Considering the vast information that has been presented, and based on it, nutritional guides can be created for patients who are often lacking in awareness and scientifically correct information regarding the vital role of nutrition in this disease. Along with patients, the clinicians are the direct beneficiaries, helping them to have a better view of the mechanisms and interactions of the nutrients with each other, but also with the medication associated with NAFLD.
